# Metal Substrate-Dependent Tribological Performance of Environmentally Acceptable Ester–PAO Lubricant Blends

**DOI:** 10.1021/acs.langmuir.6c02027

**Published:** 2026-07-08

**Authors:** Nuria Espallargas, Kastriot Ademi, Wahyu Wijanarko

**Affiliations:** The Norwegian Tribology Center, Dept. Mechanical and Industrial Engineering, Norwegian University of Science and Technology − NTNU, Richard Birkelands vei 2b, Trondheim 7034, Norway

## Abstract

Environmentally acceptable lubricants (EALs) are increasingly required in high-risk oil-to-sea interfaces, yet their boundary lubrication performance remains difficult to predict because it depends strongly on both lubricant chemistry and metal substrate reactivity. In this work, we compare the tribological response of stainless steel (AISI 316L) and bearing steel (AISI 52100) lubricated with a low-viscosity polyalphaolefin (PAO4), a polar pentaerythritol polyol ester (PE), and their 1:1 blended base oil, formulated with an environmentally acceptable ionic liquid, tributyl­(ethyl)­phosphonium diethyl phosphate (PEP), and with ZDDP as a benchmark additive. Ball-on-disc tests under identical boundary lubrication conditions show systematically lower friction and wear on bearing steel than on stainless steel, evidencing a strong substrate dependence. On stainless steel, a Cr-rich passive film limits additive-driven tribochemistry, and wear is therefore governed by base-oil polarity and additive-base lubricant competition for surface sites, which promotes abrasion when polarity increases. In contrast, the Fe-dominated chemistry of bearing steel enables tribochemical film formation, yielding lower wear across formulations. PEP delivers the most robust antiwear response, including cases of no measurable wear, and its performance is controlled by both adsorption and dispersion state: in PAO4, PEP forms an emulsion that deposits a thick viscoelastic layer suppressing wear but increasing friction, whereas in the base oil blend and PE, reduced emulsion stability and stronger surface competition shift the response toward phosphate-rich tribofilms and abrasive contributions. QCM-D reveals stronger/less reversible adsorption for PEP than for ZDDP, while ToF-SIMS confirms phosphorus-rich films as central to PEP protection and mixed P/S chemistry for ZDDP on Fe-reactive surfaces. These findings underline that the EAL formulation must be codesigned with substrate chemistry and base lubricant polarity to ensure reliable boundary performance.

## Graphical abstract

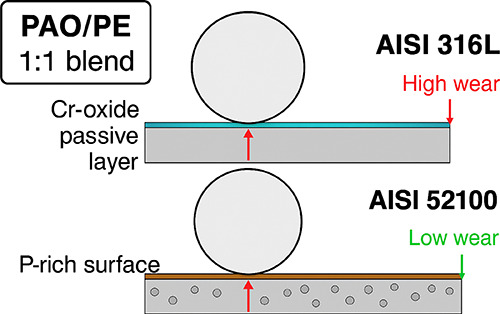

## 1 Introduction

Petroleum-based lubricants are under pressure in the future lubricant market due to their low biodegradability and toxicity.^1,2^ Along with environmental risks, the dependence of conventional lubricants on nonrenewable fossil-derived resources is another concern affecting their long-term sustainability.^3^ Avoiding their use is difficult due to their broad market presence and demonstrated performance, making them a big challenge for lubricant formulators in research and industry. The low biodegradability and high toxicity are even more concerning in components, such as in oil–water interfaces in marine applications, where the risk for leakage is very high.^4^ In addition to oil leaks, accidental, intentional, and illegal lubricant spills in seawater destroy the aquatic ecosystem. Consequently, in late 2013, the Vessel General Permit (VGP) from the US Environmental Protection Agency (EPA) announced restrictions on the type of lubricants used for nonrecreational vessels longer than 24 m operating on the US coast. VGP 2013 enforces the use of environmentally acceptable lubricants (EALs) in the oil-to-sea interfaces of those vessels.^5^ EALs are chemical blends with high biodegradability, low toxicity to aquatic organisms, and a low bioaccumulation potential. The EALs market is small due to their high production costs and, in some cases, their not-fairly reported suboptimal tribological performance resulting from their quick development and implementation with little or no dedicated qualification programs.^6^ Vegetable oils, synthetic esters, glycols, and low-viscosity polyalphaolefins (PAOs) are base fluids that fulfill the requirements for EAL formulations. Among those, synthetic esters are the most deployed base fluids for EALs.^7^

Synthetic esters derive their polarity from the two oxygen atoms in their ester group, responsible for their biodegradability and high solubility of polar lubricant additives. Additionally, the ester group has an affinity for metal surfaces, complementing, in some cases, the lubricant additives’ friction and antiwear performance.^8,9^ The polar ester group interacts with the metal surface, forming strong layers in boundary lubrication conditions.^10^ However, this polarity can also be a disadvantage as it competes with the lubricant additives for surface sites, thereby reducing the additives’ activity.^11,12^ Polyalphaolefins (PAOs) are synthetic hydrocarbons synthesized by oligomers of α-olefins, such as 1-decene.^13^ Low viscosity PAOs have a kinematic viscosity of 2–10 cSt at 100 °C.^14^ PAOs have outstanding properties, such as a high viscosity index, oxidation and hydrolytic stability, and low pour points.^15^ In addition, the cost of PAOs is lower than that of synthetic esters.^16^ Blending these base fluids is a commonly used strategy for formulating lubricants with enhanced properties.^17,18^ In the case of EALs, this can result in lower prices than the synthetic esters currently on the market.^19^ In addition, as demonstrated in our previous work, blending synthetic esters with low-viscosity PAO reduces the competition with additives and improves the solubility of highly polar additives such as ionic liquids.^20^

Expanding on the base oil blend strategy for formulating EALs, this paper evaluates the effect of the substrate material microstructure and chemical composition on the tribological performance of blends of synthetic esters and low-viscosity PAO as base fluids and environmentally acceptable additives. Stainless steel (AISI 316L) and bearing steel (AISI 52100) are the materials used in this work due to their wide range of tribological applications. AISI316L is a low-carbon austenitic stainless steel, and AISI 52100 is a high-carbon chromium-alloy steel known for its high strength due to the dispersion of spheroidal chromium carbide. Polyalpheolfin (PAO4), pentaerythritol polyol ester (PE), and their 1:1 blend were used as base fluids, while the ionic liquids tributyl­(ethyl)­phosphonium diethyl phosphate (PEP) and zinc dialkyl dithiophosphate (ZDDP) were used as lubricant additives. ZDDP was used as a reference additive. PEP already showed promising results as an alternative to ZDDP when tested with AISI316L.^20^ This paper thus studies the tribological performance in bearing steel since the metal substrate chemistry and microstructure are crucial for the lubricant performance.

## 2 Experimental Protocol

### 2.1 Materials

All base lubricants and additives in this work were selected for their compliance with environmental acceptability, except for ZDDP. Low-viscosity polyalphaolefin (PAO4) and pentaerythritol polyol ester synthesized from a mixture of octanoic, heptanoic, and 2-ethylhexanoic acids (PE) have been used as base fluids. These base fluids are fully nonpolar and polar, respectively. Chevron Philipps Chemicals (USA) kindly provided PAO4, while KAO Chemicals Europe (Germany) provided the synthetic ester. The ionic liquid additive used is tributyl­(ethyl)­phosphonium diethyl phosphate (PEP) (≥96.0%, 384.48 g/mol) purchased from VWR. Primary zinc dialkyl dithiophosphate (ZDDP) is used as reference additive, provided by Lanxess (USA). All chemicals are used as received without any further purification.

Tables 1 and 2 summarize all chemical substances used for formulating the lubricants, along with their chemical structure. Marvin software (ver. 24.1.3, Chemaxon) was used for drawing, displaying, and characterizing chemical structures. The 1:1 blended base oil is prepared by mixing PAO4 and PE in an equal ratio (1:1) by weight, and the formulated lubricants are prepared by mixing additive in the base fluid. Magnetic stirring at 40–60 °C and 500 rpm for 2–3 h is used until a clear and stable solution is formed. PEP is an ionic substance with very short organic chains, and it thus formed emulsions in the nonpolar PAO4 and in the 1:1 blended base oil. A concentration of 0.1 wt % PEP was soluble in the 1:1 blended base oil thus, to test fully soluble mixtures, the concentration of PEP was reduced in the 1:1 blended base oil. The rest of the lubricants are kept at 1 wt % additive concentration.

**Table 1:**
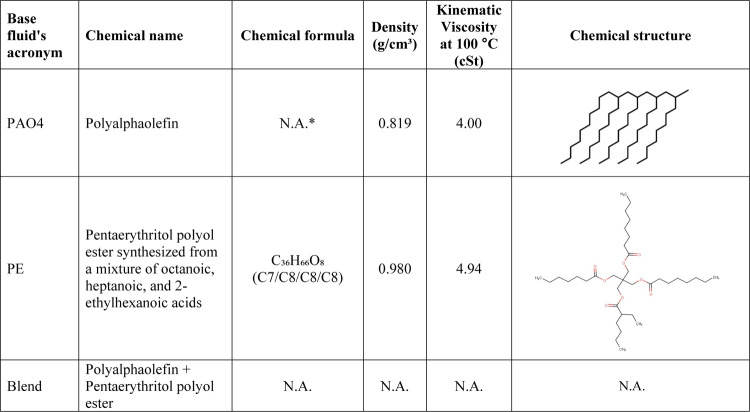
Chemical Formula, Density, and Chemical Structure of the Base Fluids Used in This Study^a^

a The hydrocarbon structure of PAO4 is not determined, since PAOs are the result of the oligomerization of linear α-olefins with a variable number of C in the alkyl chain.

**Table 2:**
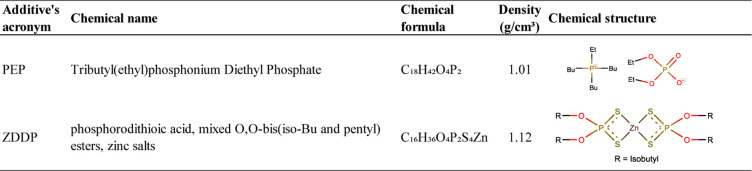
Chemical Formula, Density, and Chemical Structure of the Additives Used in This Study

As-received stainless steel AISI 316L and bearing steel AISI 52100 were used as testing materials. The AISI 316L samples were purchased from Smith Stål in Trondheim (Norway), while AISI 52100 was purchased from Basedo Steel GmbH (Germany). All samples were purchased as discs of 30 mm × 6 mm. A diamond disc, MD-Piano 80, 120, and 220 were used to grind, and diamond suspensions of 9, 6, and 3 μm on an MD-nap pad were used to polish the samples in an automatic polishing machine from Struers to mirror finishing. The polished samples were ultrasonicated for 5 min in ethanol and then air-dried. The surface roughness of the samples, measured using a Mitutoyo Surftest SJ-301 stylus profilometer, is 0.090 ± 0.003 μm.

### 2.2 Testing and Characterization Methods

A Hanna Instruments HI-88713 turbidimeter was used to test the stability and turbidity of the formulated lubricants. The stability tests were run for at least 2 h and are expressed as formazin nephelometric units (FNU). The formulated lubricants were stored for several weeks after testing to evaluate their stabilities. All formulated lubricants remained stable, even after several weeks of storage. The only lubricant forming an emulsion was PAO4 with 1 wt % PEP due to the very large difference in polarities and the short alkyl chains in PEP.

The tribological performance of the formulated lubricants was tested by using a unidirectional ball-on-disc tribometer (Rtec MFT-5000) under boundary lubricating conditions. The testing conditions included a normal load of 20 N, resulting in a contact pressure of 1.96 GPa, 30 rpm, a track diameter of 10 mm at room temperature, and a total sliding distance of 30 m. Alumina balls (Precision Ball and Gauge Co. Ltd., USA) of 6 mm diameter were used as counterpart material. All tests were repeated at least two times for reproducibility. Steel disks were ultrasonicated in ethanol before and after the test. All test repetitions were considered for analyzing the results. To demonstrate the reliability and reproducibility of the results, Table S1 in the Supporting Information discloses all friction and wear results including standard deviations.

An Alicona Infinite Focus Microscope (IFM) and MountainMap metrology software were used for wear quantification. Wear volume was measured at four distinct points along the wear track for each sample, and the average value was then computed. The specific wear rate (SWR) was calculated using Archard’s equation:

$$\mathrm{SWR}=\frac{V}{N\cdot S}$$

where SWR is the specific wear rate (mm^3^/N·m), *V* is the volume loss (mm^3^), *N* is the normal load (N), and *S* is the sliding distance (m).

A Helios G5 Plasma Focus Ion Beam (PFIB) was utilized for cross-sectional, scanning transmission electron microscopy (STEM), and time-of-flight secondary ion mass spectrometry (ToF-SIMS) analyses. To protect the surface from damage during milling for cross-sectional and STEM analyses, a 30 × 4 × 1 μm protective layer was deposited on the wear track. Carbon sources were used for electron deposition, while tungsten (W) was used for ion beam deposition. Electron deposition was done at 5 kV and 13 nA. Ion beam deposition was at 8 kV and 5 nA. A voltage of 30 kV and a current of 60 nA were used for the milling process. The cross section was cleaned under a voltage of 30 kV and a current of 60 nA and 15 nA. A ToF-SIMS detector from Thermo Fisher Scientific was used to investigate the wear tracks’ chemical composition. Two protective layers were deposited on different areas inside the wear surface for positive and negative ToF-SIMS analyses. The analysis consists of xenon primary ions with a current of 0.10 nA, a voltage of 5 kV, a pulse width of 1000, and a pulse frequency of 1 × 10^5^ Hz. The range of measured mass (*m*/*z*) was between 1 and 177. Positive and negative ion samples were collected from a 25 × 25 μm^2^ area.

A quartz crystal microbalance with dissipation (QCM-D) supplied by Biolin Scientific (Sweden) was used to study the adsorption of lubricants. 0.2 Hz is the resonance sensitivity of the instrument on liquid, the dissipation energy is 1 × 10^–7^, and the mass sensitivity is ≤1 ng/cm^2^. Stainless steel-coated and iron-coated AT-cut quartz crystals/sensors with a fundamental resonance frequency of 5 MHz from QSense (Biolin Scientific) were used. Experiments were performed at a 50 μL/min flow rate using a peristatic pump. Overtone numbers were 1, 3, 5, and 7, corresponding to 5, 15, 25, and 35 MHz frequencies. The QCM-D sensors were cleaned by immersing them in 2% Hellmanex solution for 10 min and rinsing them with acetone, distilled water, and ethanol followed by drying with nitrogen gas and finally for 10 min with a UV ozone cleaner. The QCM-D experiments consisted of running the plain base fluids for 7 min to obtain a stable baseline and then injecting the lubricant for 15 min to measure the adsorption behavior of the additives. The measurement was completed by switching back to the plain base fluid to remove the remaining adsorbed species and measure the mass of the strongly adsorbed additive molecules. Experiments were repeated at least twice to check the repeatability of the results.

All tests in this work were performed at room temperature and ambient humidity conditions in the laboratory.

## 3 Results

### 3.1 Friction Results

The results of the coefficient of friction (CoF) for the tests performed on stainless steel (SS) and bearing steel (BS) are listed in Figure 1. On SS, the CoF of PAO4 alone is 0.330, that of PAO4-PEP is 0.131, and that of PAO4-ZDDP significantly reduces friction to 0.101. In the case of PE, plain PE shows a CoF of 0.123, while PE–PEP has the lowest value of 0.116 and PE-ZDDP is 0.124. The PAO4-PE base oil blend shows lower friction than that in plain PAO4 and PE, confirming an improvement with respect to the base fluids alone. The plain base oil blend resulted in a friction of 0.111. Blend-ZDDP and blend-PEP show the lowest coefficients of friction, 0.104 and 0.108, respectively.

**Figure 1:**
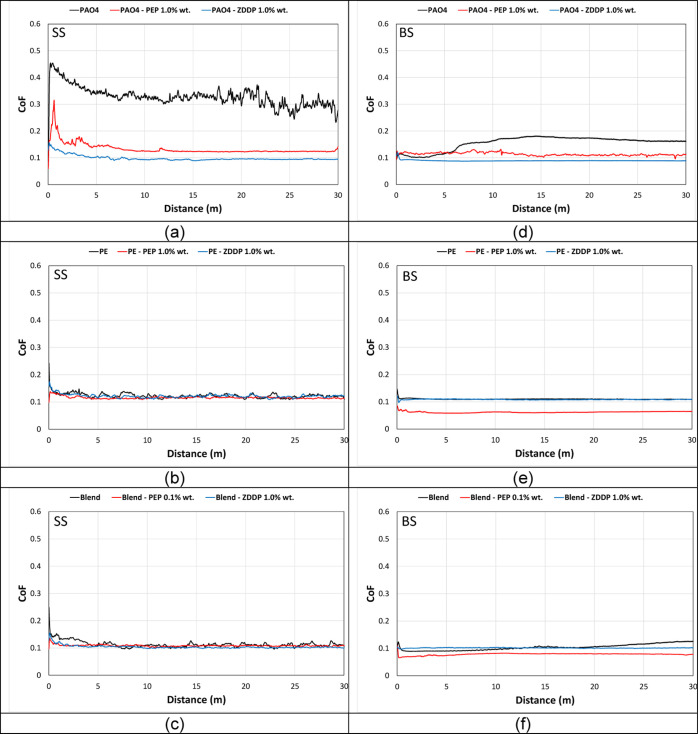
Coefficient of friction evolution in stainless steel 316L (a–c) and in bearing steel 52100 (d–f).

Friction is generally lower and smoother for the bearing steel (BS), with plain PAO4 showing a significantly lower friction coefficient (0.159) than stainless steel (0.33). Interestingly, the ionic liquid (PEP) systematically shows the lowest friction values when ester is present. However, PAO4-PEP shows the highest friction value among the additives (0.114). Plain PE shows a coefficient of friction of 0.110 followed by PE-ZDDP (0.112). The lowest friction values are found for PE–PEP (0.064) and blend-PEP (0.078). The base oil blend PAO4-PE alone shows a coefficient of friction of 0.106 followed by blend ZDDP (0.101).

### 3.2 Wear Results

The results of the wear expressed as specific wear rate (SWR) for the tests performed on stainless steel (SS) and bearing steel (BS) are shown in Figure 2. In SS, the SWR of PAO4 alone is 4.62 × 10^–5^ mm^3^/Nm, while for PAO4-ZDDP, it is 2.63 × 10^–5^ mm^3^/Nm. No measurable wear was observed for PAO4-PEP. In the case of PE, plain PE shows an SWR value of 7.17 × 10^–5^ mm^3^/Nm, while PE-ZDDP shows the lowest value of 4.01 × 10^–5^ mm^3^/Nm followed by PE–PEP with 4.67 × 10^–5^ mm^3^/Nm. A similar situation is found for the plain base oil blend, where the SWR is 5.32 × 10^–5^ mm^3^/Nm. Blend-ZDDP shows the lowest wear rate (1.55 × 10^–5^ mm^3^/Nm) followed by blend-PEP with 2.74 × 10^–5^ mm^3^/Nm.

**Figure 2:**
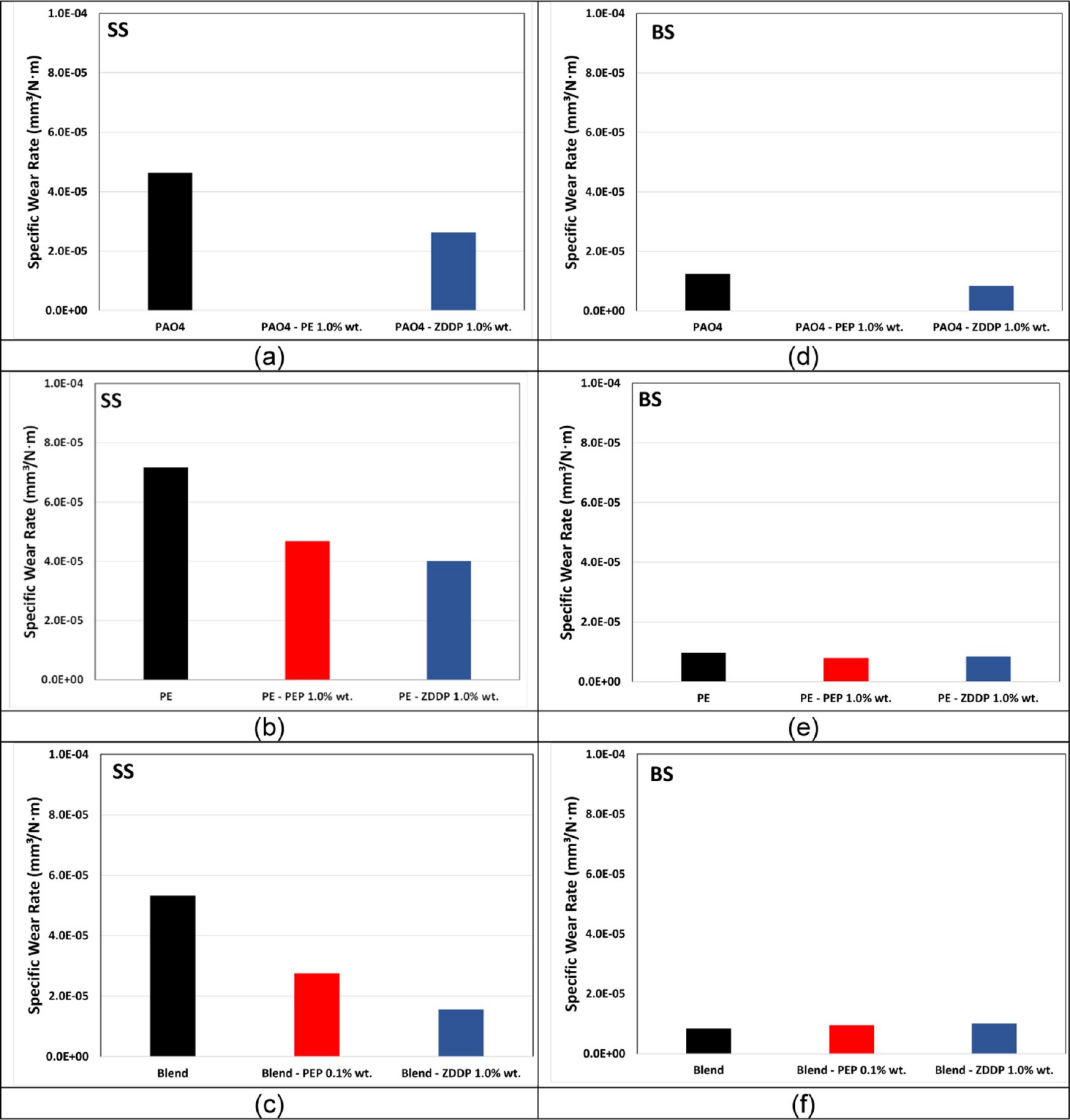
Specific wear rate evaluation in stainless steel 316L (a–c) and in bearing steel 52100 (d–f).

The wear of the lubricants tested on BS is significantly lower than that of SS, as can be clearly seen in Figure 2. The plain PAO4 shows an SWR of 1.24 × 10^–5^ mm^3^/Nm, and PAO4-PEP shows the best performance with no measurable wear followed by PAO4-ZDDP with 8.31 × 10^–6^ mm^3^/Nm. In the case of PE, the lowest wear is from PE–PEP and PE-ZDDP with 7.96 × 10^–6^ and 8.37 × 10^–6^ mm^3^/Nm, respectively, while plain PE has 8.35 × 10^–6^ mm^3^/Nm. A different situation is observed in the case of the base oil blend, where all additives show higher wear than that of the plain base oil blend. The highest wear is found for blend-ZDDP with 1.01 × 10^–5^ mm^3^/Nm, while the plain base oil blend has 8.35 × 10^–6^ mm^3^/Nm.

### 3.3 Adsorption Studies of Lubricant Additives on Stainless Steel and Iron Surfaces

The quartz crystal microbalance (QCM) is a technique used to measure the mass in nanograms of substances adsorbed on the surface of a quartz (or coated with other material) sensor. The test measures the resonance frequency, which is then converted to mass by using Sauerbrey’s equation:

$$\Delta f=-\frac{2{f_{0}}^{2}}{A\sqrt{\rho_{q}\mu_{q}}}x\Delta m$$ 1

where *f*_0_ is the sensor’s fundamental frequency, ρ_q_ is the density of the quartz plate (ρ_q_ = 2.648 g/cm^3^), μ_q_ is the shear modulus of quartz (μ_q_ = 2.947 × 10^11^ g/cm s^2^), and *A* is the area of the sensor.

Important to note is that QCM results are complementary to tribological testing and thus offer mechanistic understanding of adsorption phenomena rather than a direct representation of tribofilm formation or performance under boundary lubrication. The QCM-D results of this work provide information about adsorption strength and viscoelastic properties under well-controlled conditions, but they do not reproduce the high pressure, shear, and tribochemical conditions present in real sliding contacts.

Figures 3 and 4 show the frequency and dissipation results obtained for stainless steel and iron-coated sensors. In the case of stainless steel, stronger adsorption on PAO4 is observed for PEP, showing ongoing (nonstop) adsorption reaching 95 Hz during the time of the experiment and remaining on the surface after rinsing with plain PAO4 (50 Hz), indicating strong (chemisorption) surface adsorption (Figure 3a). PEP forms an emulsion in PAO4, resulting in complex, non-Sauerbrey behavior, with droplets depositing on the sensor. The stability of the emulsion can be measured by looking at dissipation (Figure 4a). High dissipation values are found for PAO4-PEP, resulting in a highly viscoelastic layer. This suggests a strong droplet-surface interaction stabilizing the emulsion against coalescence. PAO4-ZDDP shows adsorption with a frequency drop of 31 Hz, and after rinsing with plain PAO4, the additive slightly remains on the surface, as the frequency does not return to zero (5.7 Hz).

**Figure 3:**
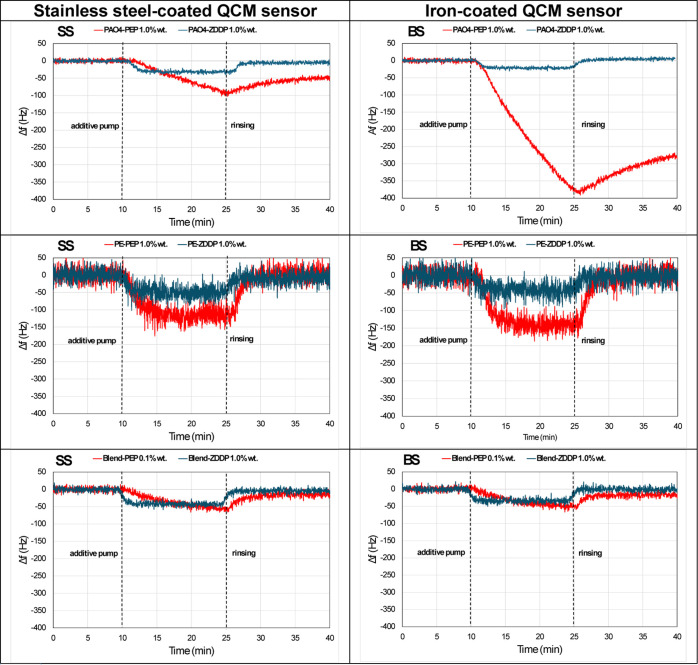
Frequency evolution during QCM-D testing on the stainless steel sensor (a–c) and Fe sensor (d–f). The base lubricant with the additives is pumped after 10 min, and after 25 min, the plain base lubricant is pumped in again (rinsing).

**Figure 4:**
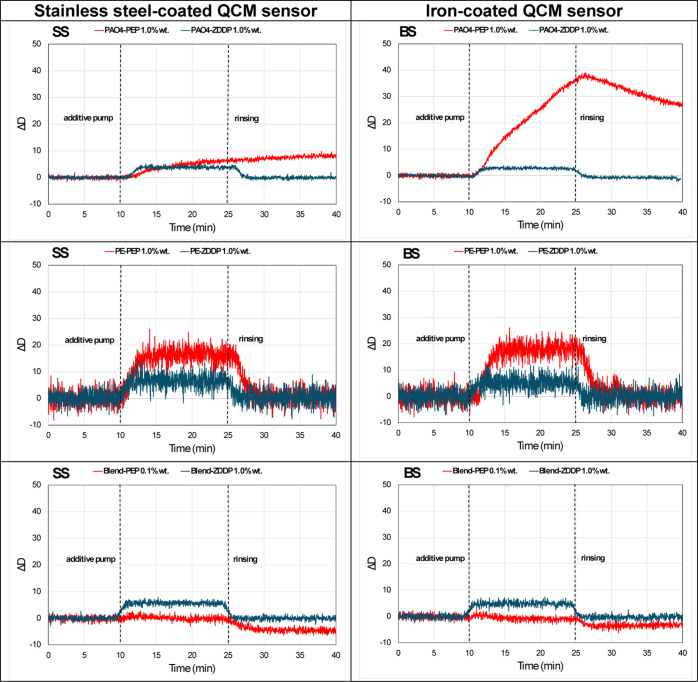
Dissipation evolution during QCM-D testing on the stainless steel sensor (a–c) and Fe sensor (d–f). The base lubricant with the additives is pumped after 10 min, and after 25 min, the plain base lubricant is pumped in again (rinsing).

In the case of the base oil blend, PEP shows ongoing adsorption reaching 60 Hz during the time of the experiment (Figure 3b) and remaining on the surface after rinsing (15 Hz). This might suggest that even at the lower concentration of 0.1 wt %, PEP still forms an emulsion. However, when looking at the dissipation and comparing it to PAO4, very low values are found, indicating the formation of a rigid layer, suggesting that the emulsion is not stable and breaks at the surface. Blend-ZDDP shows a frequency drop of 42 Hz, which remains on the surface after rinsing with plain base oil blend (4 Hz).

Table 1 discloses the kinematic viscosity of PAO4 and PE at 100 °C where very similar values for the two base fluids is found. Their kinematic viscosity at 40 °C is 16.8 and 24.8 cSt, respectively, which leads to a different viscosity index (140 for PAO4 and 126 for PE). Thus, at temperatures below 40 °C, the kinematic viscosity of PE will be higher than PAO4. QCM tests were performed at room temperature; thus, PE’s higher viscosity than PAO4 results in noise in the frequency measurements for this base lubricant. PE-PEP (Figure 3c) shows the strongest adsorption among the formulated lubricants, with a frequency drop of 114 Hz, but does not remain on the surface after rinsing with PE. In contrast, PE-ZDDP shows weaker adsorption with a frequency drop of around 49 Hz and slightly remains on the surface after rinsing with plain PE (7.9 Hz).

The tests performed for PAO4-PEP on the iron sensor also show the strongest adsorption with typical emulsion behavior, with ongoing (nonstop) adsorption reaching 385 Hz during the time of the experiment (Figure 3d). PAO4-PEP also shows irreversible adsorption, remaining on the surface after being rinsed with PAO4 (280 Hz). PAO4-ZDDP shows a significantly lower adsorption, with a frequency drop of 21 Hz, and does not remain on the surface after being rinsed with plain PAO4.

In the case of the base oil blend, blend-PEP also shows an ongoing (nonstop) adsorption, reaching 52 Hz during the time of the experiment (Figure 3e) and remaining on the surface after rinsing at 16 Hz, indicating emulsion behavior as in stainless steel. Blend-ZDDP shows less adsorption with a frequency drop of 34 Hz and does not remain on the surface after rinsing with a plain base oil blend.

Noise in the frequency measurements is observed in the case of PE due to its high viscosity. PE-PEP shows the strongest adsorption with a frequency drop of 140 Hz, leaving the surface after being rinsed with plain PE (Figure 3f). PE-ZDDP shows lower adsorption with a frequency drop of 40 Hz and remains on the surface after being rinsed with plain PE (4 Hz).

## 4 Discussion

Among many factors, the friction and wear performance of lubricants is affected by the chemistry of the metal substrate material they are tested on. Stainless steel (AISI SS316L) is a low-carbon steel material with high content of chromium and nickel, while bearing steel (AISI 52100) is a chromium-alloy steel material with high-carbon content (Table 3). The stainless steel chosen for this work was in as-received conditions with an austenite microstructure, while the bearing steel was in as-received conditions with dispersion of spheroidal carbides in a matrix of ferrite. The lubricants’ functional mechanisms are additives’ adsorption on the metal surface, followed in specific conditions by tribofilm formation, normally under boundary lubricating conditions.^21,22^ Adsorption can be physical or chemical, while tribofilm formation is a chemical reaction between the lubricant chemistry and the metal surface mediated by the mechanical action. Tribofilms are typically nanometer-thick layers that effectively separate the sliding surfaces, thereby reducing wear.

**Table 3:** Chemical Composition of Stainless and Bearing Steels (Data Collected from *MatWeb* Database^23^)

Element (wt %)	Fe	Cr	Ni	Mo	Mn	Si	P	S	C	Cu
**SS 316L**	65.61	17.5	11.5	2.20	2.0	1.0	0.045	0.015	0.03	0.10
**BS 52100**	96.41	1.55	0.25	0.10	0.35	0.015	0.025	0.015	0.99	0.30

### 4.1 Effect of Metal Composition on Wear

The chemistry/composition of the steel plays a crucial role in its tribological performance due to its reactivity with additives, with this effect being more relevant for wear than for friction. Interestingly, thick tribofilms formed on bearing steel do not significantly affect the frictional performance of lubricants, but they rather provide better wear protection, as it has been reported in the literature.^20,23–29^ In this work, this effect is also supported by the tribofilms formed on bearing steel as indicated from the wear track surface chemistry analysis by ToF-SIMS (see the Supporting Information) and by STEM imaging (Figure 5). Surface chemistry is thus crucial in controlling wear performance, as it involves chemical interactions between the tribosurface and the lubricant.^27^ The elemental composition of the two steels in this work is different enough to evaluate their reactivity toward the different additives. For instance, the availability of elements like Cr (metallic vs carbide in stainless steel and bearing steel, respectively) is an important parameter to study.

**Figure 5:**
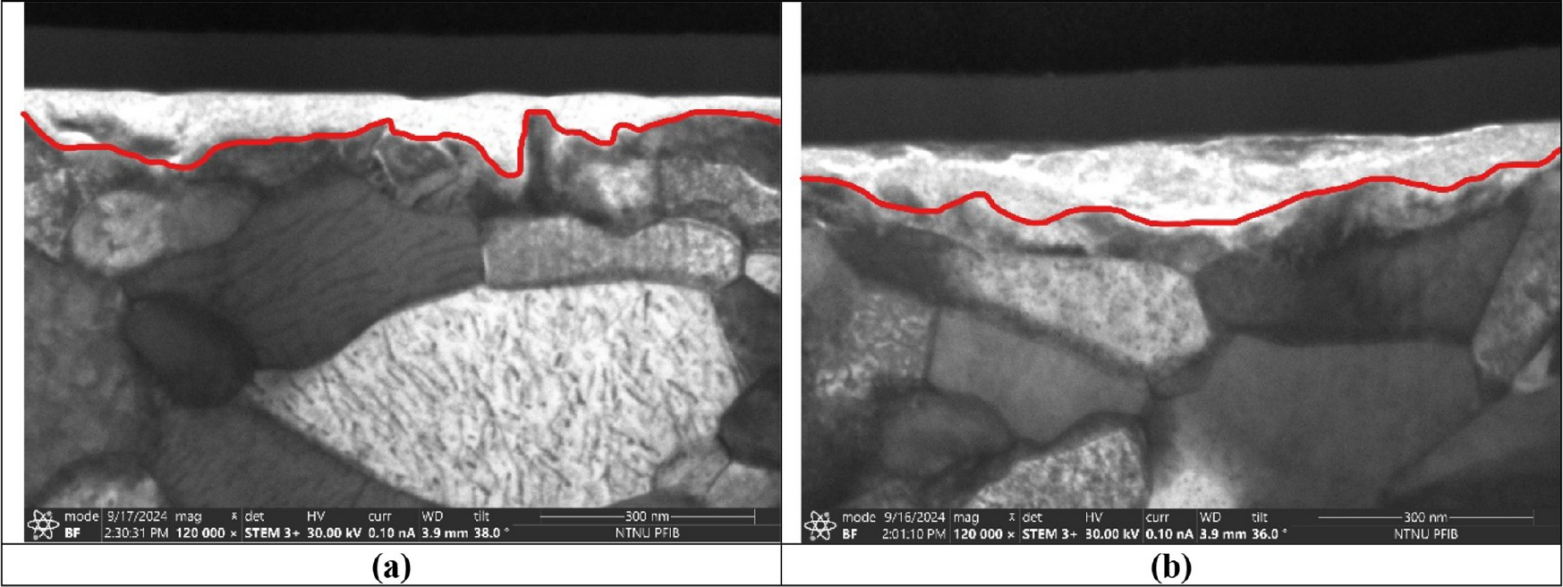
STEM cross-sectional images of the bearing steel after testing blend-PEP (a) and blend-ZDDP (b). The red lines show the tribofilm formed.

When comparing the wear rates of the lubricants in the two materials, significantly lower wear is systematically found in bearing steel (Figure 2). The differences are three times lower in the case of PAO4 and up to 10 times lower in the case of PE and the base oil blend, which is highly correlated to the steels’ chemical composition (Table 3) and with the wear track surface chemistry (see the Supporting Information). Stainless steel contains approximately 17 wt % elemental chromium (Cr), which provides corrosion protection through the formation of a thin passive layer (mostly chromium oxide). This layer may hinder chemical reactions between the steel and the lubricant additives. Nickel (Ni) also holds a non-negligible percentage in stainless steel (11.5 wt %) and may also affect the chemical reactivity of the steel with the lubricant additives. This leaves only around 65 wt % of iron (Fe) available in stainless steel for reacting with the lubricant additives. In the case of the bearing steel, around 97 wt % of the composition of the bearing steel is elemental iron (Fe) and chromium is present in its spheroidized form as carbide allowing for more reactivity with the lubricant additives.

#### 4.1.1 Surface Chemistry and Wear Mechanisms of ZDDP-Containing Lubricants

On stainless steel, ToF-SIMS shows the highest intensity for O^–^ and OH^–^ species in the negative spectra and the Cr^+^ is the most intense specie in the positive spectra for all base lubricants. The species representative of the heteroatoms in the additive (S^–^ and PO_2_^–^) are present in very small intensities (Figures S1–S6). This shows that the passive film chemistry dominates the wear track surface and thus, the decomposition of the ZDDP followed by tribochemical reactions can be neglected as the main driver for wear resistance. Interestingly, the wear rate (Δ*A*) seems to be much more correlated with the polarity of the base lubricant, i.e., increasing wear rates as polarity increases (Figure 6a). This might be an indication of the competition between ZDDP and the base lubricant for the metal substrate adsorption sites. Indeed, the adsorption of ZDDP measured by QCM clearly shows this effect. Table 4 summarizes the frequency drop (Δ*f*) and dissipation (Δ*D*) results of the third overtone. The frequency and dissipation values of the third overtone for a standard 5 MHz crystal provide information related to both surface adsorbed mass (Δ*f*) and viscoelastic (Δ*D*) properties of the adsorbed layers. For ZDDP on stainless steel, more mass is adsorbed on the sensor when the base lubricant polarity increases being the highest for PE (the most polar base lubricant), which also correlates with lower intensity on the O^–^ and OH^–^ maps in ToF-SIMS (Supporting Information). This agrees with the higher wear rates since the presence of more polar species like the ester at the surface will result in the oxidation of the wear debris particles (the ester mostly contains oxygen), leading to higher material loss by abrasion (higher β values, Figure 6b).

**Table 4:** Summary of Adsorption Results (Frequency and Dissipation) of ZDDP and PEP in PAO4, 1:1 Base Oil Blend, and PE on Stainless Steel (SS) and Iron (Fe)-Coated Sensors

	stainless steel			bearing steel		
	Δ*f* (Hz)	Δ*D*	after rinsing	Δ*f* (Hz)	Δ*D*	after rinsing
**PAO4-ZDDP**	–30	3.8	no ads.	–25	3.8	no ads.
**Blend-ZDDP**	–38	5.7	no ads.	–30	4.9	no ads.
**PE-ZDDP**	–50	16	no ads.	–38	5.5	no ads.
**PAO4-PEP**	<−95^a^	>5^a^	strong ads.	<−385^a^	>38^a^	strong ads.
**Blend-PEP**	–55	0	weak ads.	–52	0	weak ads.
**PE–PEP**	–114	16	no ads.	–140	18	no ads.

a Note the very different slopes in Figure 4, being steeper for the bearing steel. In addition, note that this behavior is due to the formation of an emulsion.

**Figure 6:**
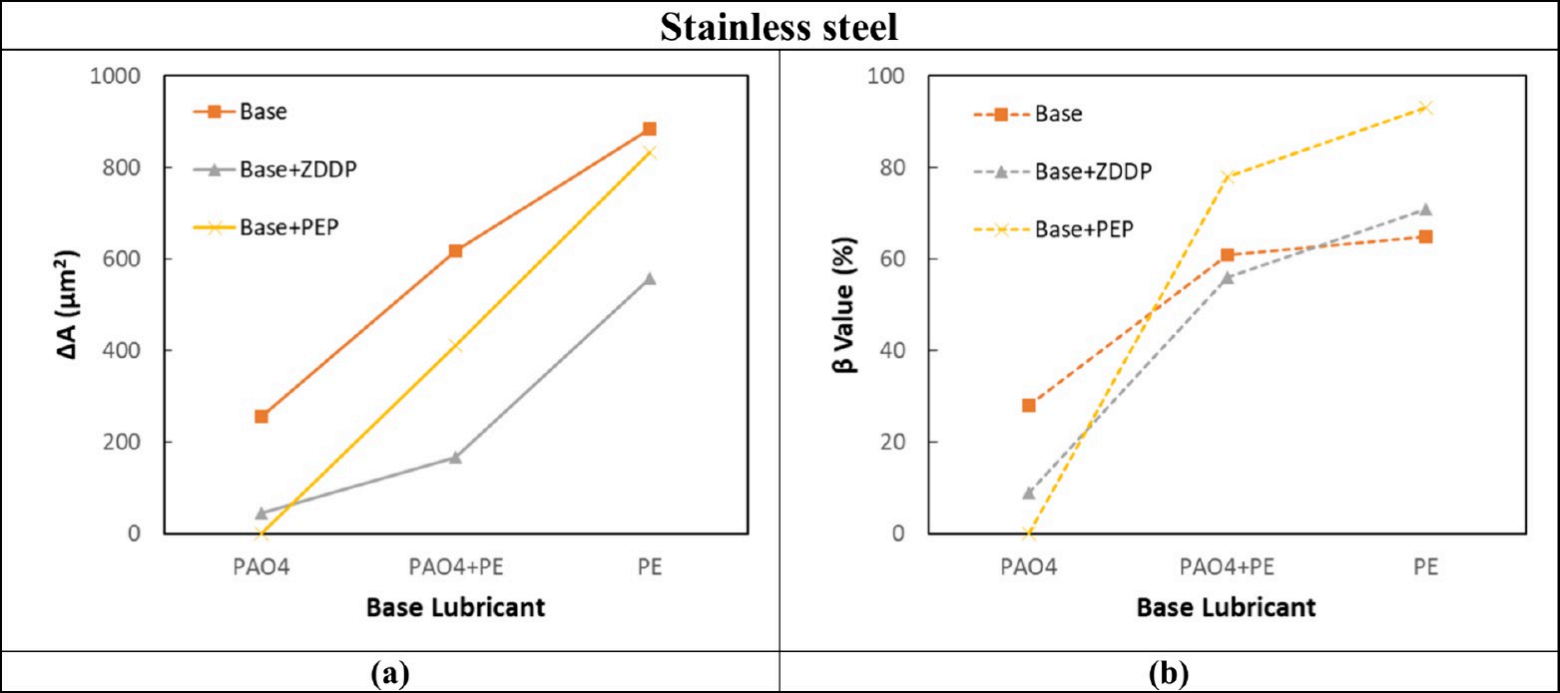
Wear detail parameters for actual material loss (Δ*A*) (a) and (b) β value in stainless steel. Δ*A* is the difference between the *A*_groove_ (material loss in the wear track) and the *A*_ridge_ (material displacement). The β value is the ratio between Δ*A* and *A*_groove_. The higher the β value, the more abrasion in the system.

On bearing steel, ZDDP behaves differently because the surface is Fe-dominated and readily supplies iron cations (Figures S7–S12) for forming Fe-polyphosphate glassy layers. In this case, tribochemistry is clearly taking place at the contact forming tribofilms (Figure 5b) and wear is thus not controlled by the polarity of the base lubricant (Figure 7). Indeed, PAO4-ZDDP wear track chemistry is PO_2_^–^-dominated (Figure 8), consistent with a phosphate-rich tribofilm that yields low wear. The lack of polarity in PAO4 allows ZDDP to occupy more surface sites that, together with the catalytic role of Fe cations at the surface, accelerate1s phosphate condensation into a thicker polyphosphate tribofilm. Indeed, the intensity of the Fe^+^ in PAO4-ZDDP by ToF-SIMS is the highest of all base lubricants (Figure 8). Using the 1:1 base oil blend as base lubricant shifts surface chemistry toward sulfur and phosphorus (with higher S) and increases wear. PE’s polarity alters ZDDP’s decomposition route and redistributes additives at the contact, yielding a more sulfide-rich tribofilm resulting in the lowest wear. In addition, in the case of plain PE, more mass is adsorbed at the surface (Table 4) promoting a continuous S-rich tribofilm minimizing abrasive debris.

**Figure 7:**
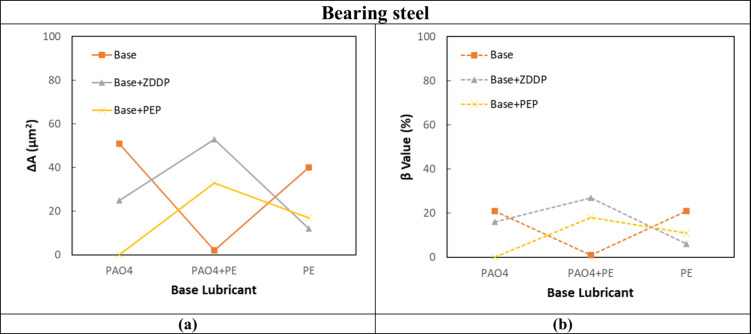
Wear detail parameters for actual material loss (Δ*A*) (a) and (b) β value in bearing steel. Δ*A* is the difference between the *A*_groove_ (material loss in the wear track) and the *A*_ridge_ (material displacement). The β value is the ratio between Δ*A* and *A*_groove_. The higher the β value, the more abrasion in the system.

**Figure 8:**
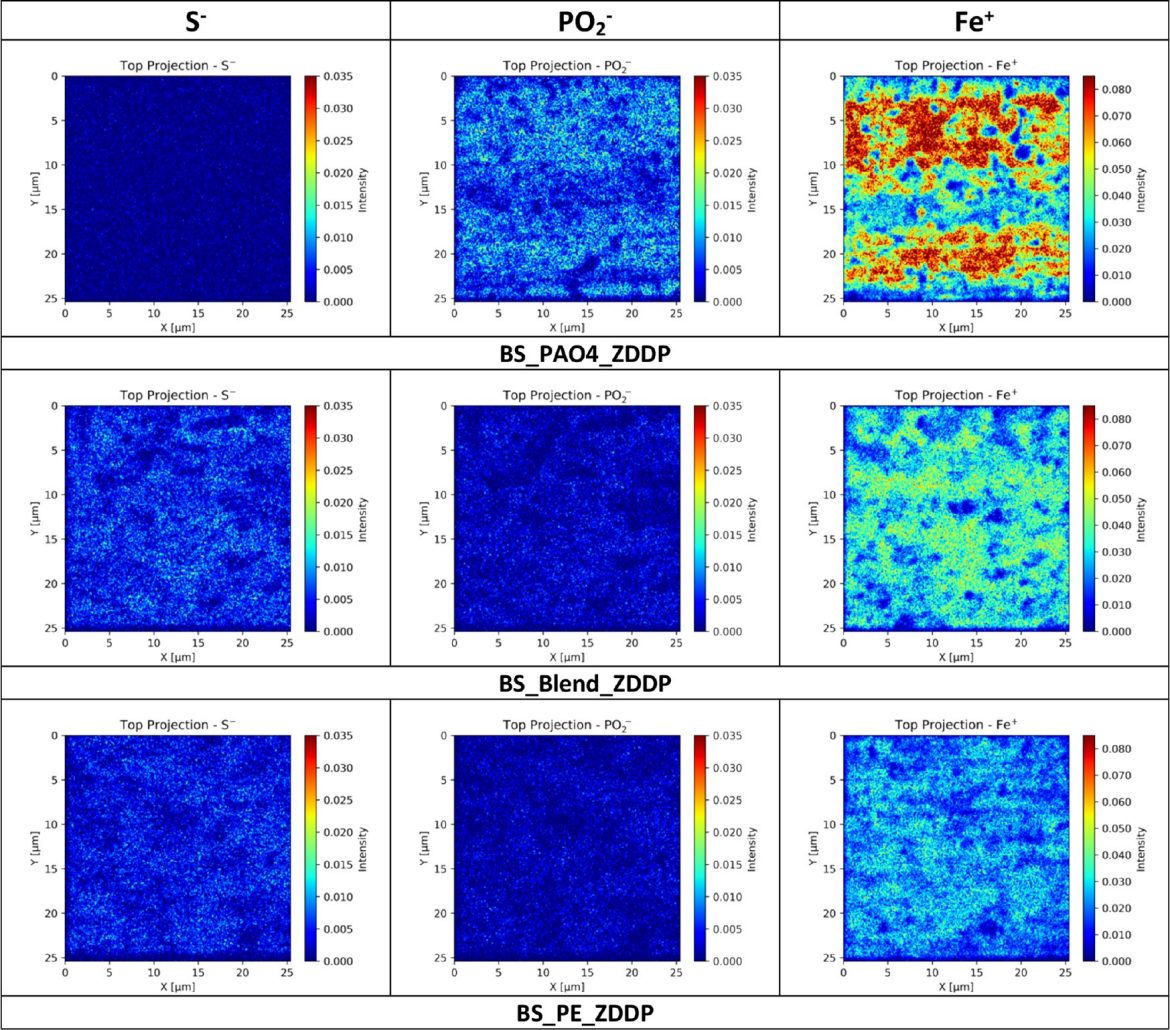
ToF-SIMS S^–^, PO_2_^–^, and Fe^+^ spectra for ZDDP in all base lubricants on the bearing steel substrate.

#### 4.1.2 Surface Chemistry and Wear Mechanisms of PEP-Containing Lubricants

The case of PEP should be considered in terms of emulsion formation in PAO4 and the 1:1 base oil blend and concentration in the base oil blend. These two parameters control the behavior of this additive, resulting in different mechanisms on each metal substrate.

On stainless steel, the same predominance of the passive film as in the case of ZDDP is found on ToF-SIMS, with the highest intensity for O^–^ and OH^–^ species in the negative spectra and Cr^+^ in the positive spectra for all base lubricants (Figures S13–S18) followed by the heteroatom present in the additive (PO_2_^–^). Indicating once again the little tribochemical action of the additive on the surface. The PO_2_^–^ maps show the highest intensity for blend-PEP and the lowest for PAO4-PEP (Figure 9), which agrees with the emulsion formation in PAO4 and the additive-base lubricant competition in the base oil blend and PE. The wear response diverges strongly (Figure 6): PAO4-PEP yields zero wear (β ≈ 0% indicating pure ploughing), while blend-PEP and PE–PEP suffer abrasion-dominated wear with β values of 78% and 93%, respectively. To understand this behavior the effect of the emulsion formation on friction evolution (Figure 1) and metal surface adsorption (Table 4) are to be taken into the discussion. In PAO4, during the running-in period at the beginning of the sliding process (Figure 1a), metal is removed and friction is high. As the emulsion builds up at the contact, PEP strongly adsorbs at the nascent metal surface sites (Figure 3a) abundantly and strongly. That adsorbed layer is abundant and highly viscoelastic (Figure 4a and Table 4, highest Δ*D*) and it can be assumed that the emulsion droplets are able to efficiently separate the sliding surfaces building up a fluid film that completely suppresses wear. Indeed, ToF-SIMS barely shows any PO_2_^–^ at the metal surface (Figure 9) confirming that the PEP emulsion droplets have not undergone any tribochemical interaction with the metal surface. Adding PE (a polar polyol ester) to PAO4 destabilizes the emulsion (Table 4, lowest Δ*D*) and introduces a competing mechanism with PEP for the metal surface sites favoring the formation of phosphate-rich networks/precipitates that are thick but brittle. Indeed, higher intensity of Fe^+^ and PO_2_^–^ are found for blend-PEP and PE–PEP (Figure 9), indicating that a more ready interaction of the additive with the metal surface is taking place. Under shear, these discontinuous phosphate regions wear out, exposing the nascent metal substrate releasing debris and shifting the wear mechanism to more abrasive and increasing wear rates. The surface competition mechanism can be seen in the changes in adsorption (Figures 3 and 4), where frequency and dissipation go from continuous decrease and growth (PAO4) to constant low values (base oil blend and PE), resulting in weak or no adsorption after rinsing with the base lubricant.

**Figure 9:**
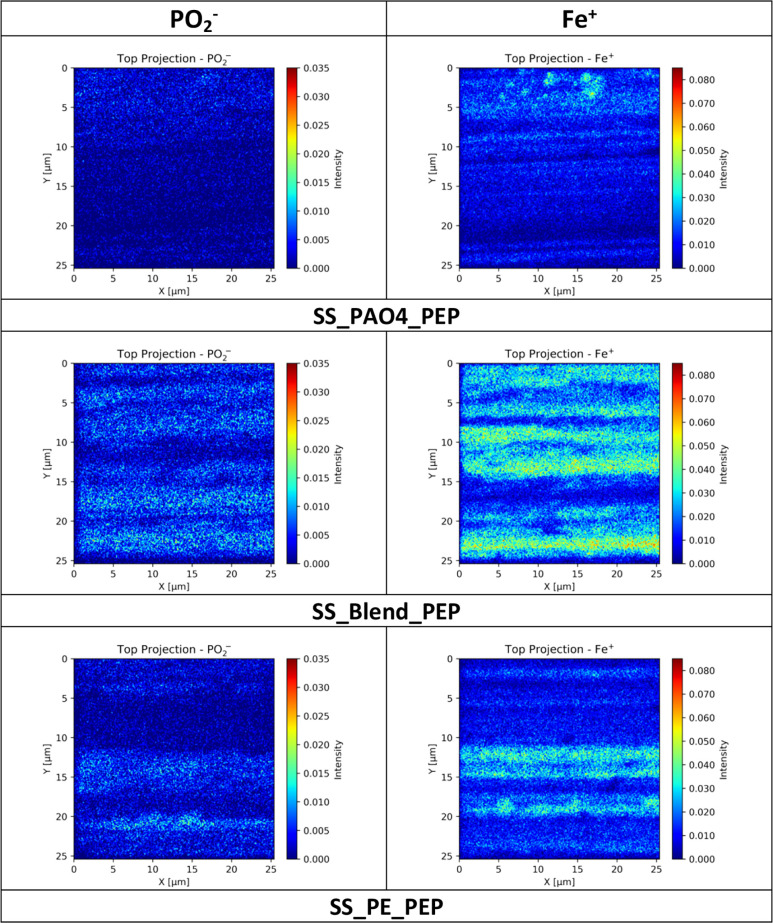
ToF-SIMS Fe^+^ and PO_2_^–^ spectra for PEP in all base lubricants on the stainless steel substrate.

On bearing steel, PEP results in higher intensity maps of Fe^+^, O^–^, and PO_2_^–^ (Figure 10). The intensity map of PO_2_^–^ is the highest for the blend-PEP. Again, the material loss of bearing steel is negligible for PAO4-PEP followed by PE-PEP and blend-PEP (Figure 7), which is due to the emulsion formation. However, PEP does not form emulsions in PE and it forms an unstable emulsion in the base oil blend; thus, the Fe-dominated microstructure supplies Fe ions during sliding allowing PEP to form iron-poly/pyrophosphate “glassy” networks that are adhesive and shear-compliant. Indeed, the wear rates are also correlated to the adsorption results (Table 4) with the highest dissipation (and frequency drop) for PAO4-PEP followed by PE-PEP and blend-PEP. In the case of PAO4-PEP, the emulsion forms a very thick viscoelastic layer that efficiently separates the contacting surfaces completely suppressing wear. However, as polarity increases, the emulsion is not present, and the additive is more readily available to interact with the metal surface. The highest polarity of PE results in a strong adsorption competition with the additive for surface sites and less PO_2_^–^ is found at the surface (Figure 10). For the base oil blend, less competition takes place and PEP reacts more readily with the metal surface resulting in higher PO_2_^–^ intensity (Figure 10).

**Figure 10:**
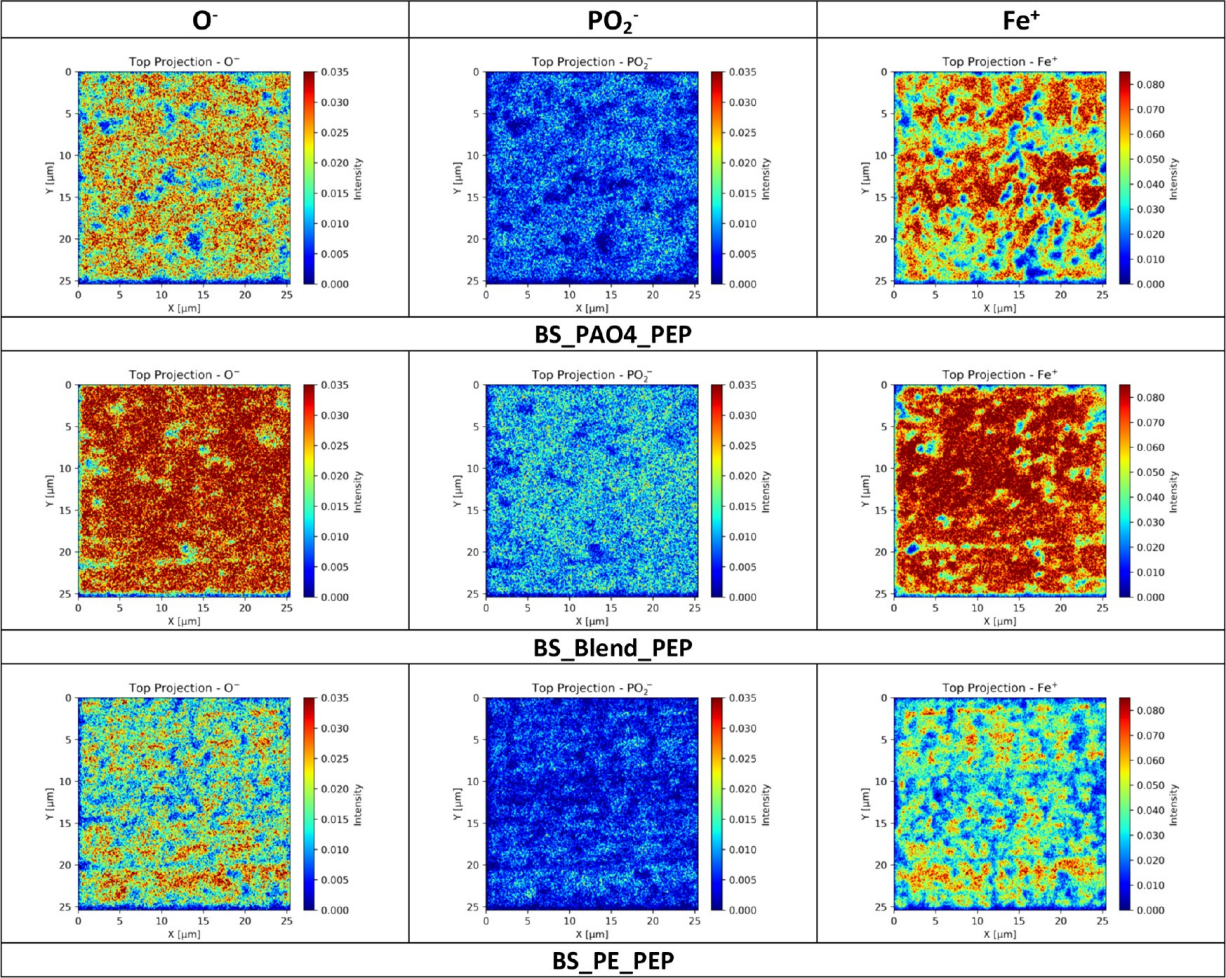
ToF-SIMS Fe^+^ and PO_2_^–^ spectra for PEP in all base lubricants on the bearing steel substrate.

### 4.2 Metal Microstructure and Additive Adsorption Effects on Friction

In addition to the wear performance achieved by the additives studied in this work, noteworthy friction differences were found (Figure 11). On stainless steel, both additives reduce friction significantly with respect to that of the base lubricant. When the ester is introduced as a base lubricant (either plain or as a base oil blend), there are no differences in friction between the plain base lubricant and the formulated version, which can be understood because of the surface adsorption competition between the ester and the additives. Comparing the frictional performance of the additives on stainless steel, a slight tendency to a friction increase with base lubricant polarity was found. For ZDDP, this tendency agrees with the dissipation increase in QCM (i.e., an increase in the viscoelasticity of the adsorbed layer), as seen in Figure 12. For PEP, this tendency is broken for PAO4-PEP because of the formation of an emulsion. The emulsion forms a highly viscoelastic fluid film at the ball-metal sliding contact, efficiently separating the moving surfaces. This highly viscoelastic layer results in high friction due to increased energy dissipation causing viscous losses due to the velocity dependency of viscosity. In the case of the blend-PEP, the emulsion formed is unstable, resulting in a rigid adsorbed layer with lower friction. When the base lubricant is fully polar (PE), the adsorbed layer is more viscoelastic and friction increases again.

**Figure 11:**
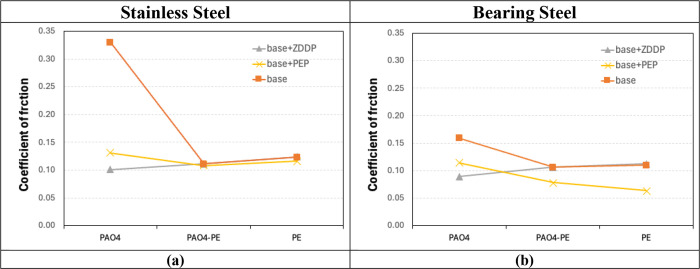
Friction detail parameters for ZDDP and PEP in all base lubricants for stainless steel (a) and bearing steel (b).

**Figure 12:**
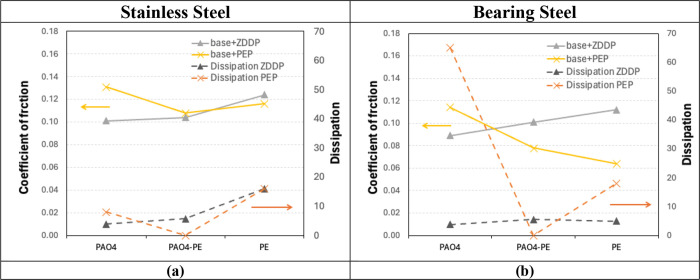
Friction and dissipation results for ZDDP and PEP in all base lubricants for stainless steel (a) and bearing steel (b).

On bearing steel, a smaller friction reduction with respect to the base lubricant is found (Figure 11), including a reduced running-in period and smoother friction evolution over time (Figure 1). Indeed, the smoothness of the friction evolution and the lack of running-in on the bearing steel are found for all lubricants. The fact that plain and formulated base lubricants show significantly better frictional performance on bearing steel can be attributed to their microstructure, which consists of spheroidal chromium carbides (dark spheres) distributed evenly across the entire microstructure (Figure 13). The dispersion of spheroidal carbides within the matrix enhances the local strength and load-bearing capacity of the material, making the bearing steel more resistant to sliding and resulting in lower friction. Indeed, the presence of these carbides contributes significantly to its mechanical response under sliding contact, even though the bearing steel used in this work is not hardened (hardness 194 HVN0.5). The low hardness of the bearing steel, lower than that of stainless steel (267 HVN0.5), demonstrates that the strengthening mechanism of the material given by the carbides is a crucial factor in its frictional performance. Indeed, this is supported by the FIB-SEM cross-sectional images (Figure 13), where no degree of recrystallization under the wear track is found for bearing steel, indicating that the shear forces during sliding did not result in plastic deformation and thus kept low friction. This work demonstrates that carbide strengthening mechanisms are key for frictional performance, whereas hardness might not be a critical factor. Therefore, friction on bearing steel does not align with the dissipation measurements (Figure 12) since the material strength and the tribochemical reactions discussed in Section 4.1 are the governing mechanisms for frictional response in this case.

**Figure 13:**
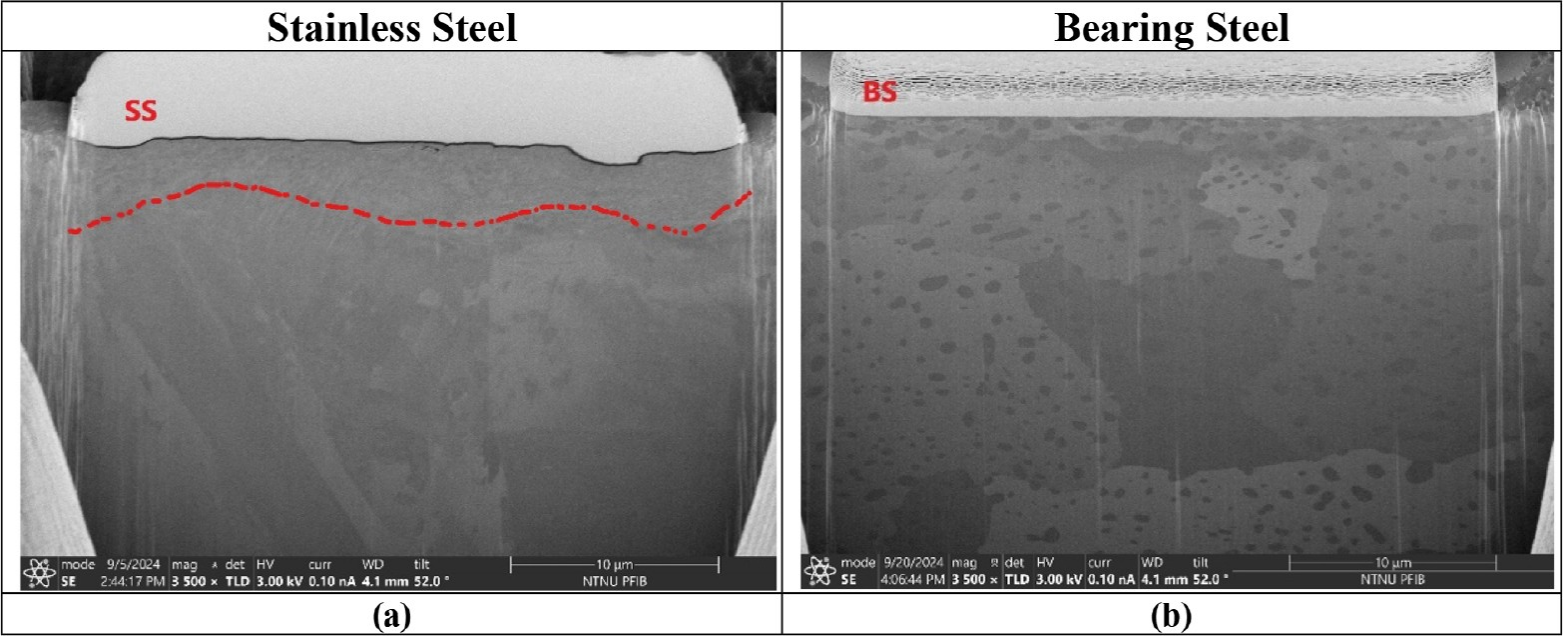
PFIB cross-sectional images for PAO4-PEP in stainless steel (a) and bearing steel (b). The red line drawn in SS shows the recrystallization region of the subsurface.

Overall, the tribological response is substrate-controlled: on AISI 316L, the Cr-rich passive film suppresses additive tribochemistry and wear is mainly dictated by base-oil polarity and adsorption competition, whereas on AISI 52100, the Fe-dominated surface promotes tribochemical film formation and generally lower wear. PEP provides the most robust protection, switching from an emulsion-derived viscoelastic separating layer in PAO4 to phosphate-rich tribofilms in the blend-PE as emulsion stability decreases and surface competition increases. QCM-D and ToF-SIMS support stronger PEP adsorption and P-rich films versus mixed P/S chemistry for ZDDP on Fe-reactive surfaces. Figure 14 schematically summarizes these mechanisms for each base-oil/additive combination on both steels.

**Figure 14:**
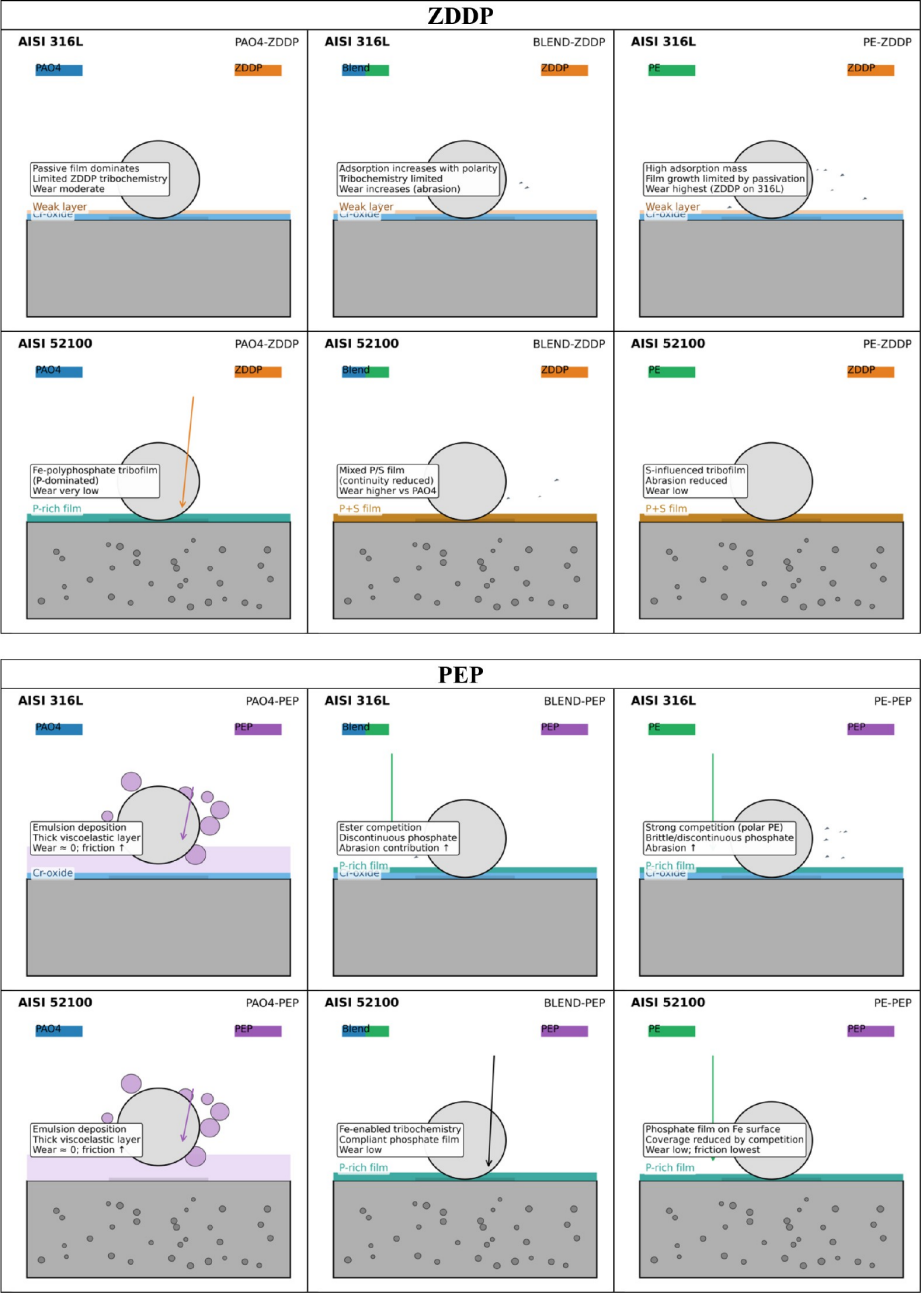
Summary of the mechanisms taking place for ZDDP and PEP on the different base lubricants and steel substrates.

## 5 Conclusions

This study shows that the tribological performance of environmentally acceptable lubricant formulations under boundary lubrication is controlled by the combined effects of metal substrate chemistry and microstructure, base oil polarity, and additive chemistry. PAO4, pentaerythritol ester (PE), and their 1:1 blended base oil were evaluated with PEP and ZDDP on stainless steel (AISI 316L) and bearing steel (AISI 52100).

AISI 52100 consistently exhibited lower friction and wear than AISI 316L, reflecting the strong influence of its Fe-rich composition and carbide-strengthened microstructure. In contrast, the Cr-rich passive layer on AISI 316L limits additive-surface reactivity, suppressing tribochemical film formation.

The results further show that wear is not determined by substrate reactivity or lubricant formulation alone but by their interplay. On AISI 52100, the availability of Fe promotes phosphate- and polyphosphate-containing tribofilms for both additives, leading to lower wear rates. On AISI 316L, where tribochemical activity is restricted, the base oil polarity and adsorption competition become more important and are associated with more abrasive wear.

The base oil polarity also strongly affects additive performance. Increasing the polarity from PAO4 to PE enhances competition for surface adsorption sites, modifies the nature of the tribofilms, and can increase abrasion, particularly on AISI 316L.

Among the two additives, PEP showed the most robust antiwear behavior. Zero measurable wear was obtained for PAO4-PEP on both steels, while in PE and in the blended base oil, the protection was associated with phosphate-rich tribofilms. By comparison, ZDDP formed more effective tribofilms mainly on the Fe-reactive bearing steel, where mixed sulfur- and phosphorus-containing tribofilms were observed.

The key novelty of this work is the demonstration that the performance of ester-PAO environmentally acceptable lubricant formulations cannot be understood from additive chemistry alone but must be interpreted together with substrate composition, microstructure, and base oil polarity. These findings provide a clearer basis for the substrate-specific design of environmentally acceptable lubricants for boundary lubricated contacts.

## Supplementary Material



## Notes

Complete set of friction and wear data sets with standard deviations for all repetitions, and additional ToF-SIMS positive and negative ion maps/spectra of wear tracks for ZDDP- and PEP-formulated lubricants in PAO4, PE, and the 1:1 PAO4-PE blended base oil on AISI 316L and AISI 52100 (PDF)

## References

[1] Singh Y. (2015). Tribological
behavior as lubricant additive and physiochemical characterization
of Jatropha oil blends. Friction.

[2] Menon K. S., Rajasekaran A. (2023). Evaluation
of tribological properties and sustainability
of bio-lubricant developed from neem seed oil for real-life application. Tribiol. Int..

[3] Rafiq M., Lv Y. Z., Zhou Y., Ma K. B., Wang W., Li C. R., Wang Q. (2015). Use of vegetable
oils as transformer
oils – a review. Renewable and Sustainable
Energy Reviews.

[4] Borras F. X., De Rooij M. B., Schipper D. J. (2018). Rheological and
Wetting Properties
of Environmentally Acceptable Lubricants (EALs) for Application in
Stern Tube Seals. Lubricants.

[5] Wodtke M., Frost J., Litwin W. (2025). Effect of
water contamination of
an environmentally acceptable lubricant based on synthetic esters
on the wear and hydrodynamic properties of stern tube bearing. Tribiol. Int..

[6] Shah R., Woydt M., Zhang S. (2021). The Economic
and Environmental Significance
of Sustainable Lubricants. Lubricants.

[7] Bayat R., Lehtovaara A. (2020). EHL/mixed
transition of fully formulated environmentally
acceptable gear oils. Tribiol. Int..

[8] Wang Y., Liang Y., Li Y., Rui W., He J., Zhao M. (2024). Synthesis, tribological properties
and oxidative stability of polyol
esters base oils containing pentaerythritol complex esters. Tribiol. Int..

[9] Wang H., Zhang C., Chen H., Yu X., Li Y., Yang K. (2024). Influence of ether group on viscosity
and film lubrication of diester
lubricants: Integrated quantitative structure–property relationship
and molecular dynamics simulation methods. J.
Mol. Liq..

[10] Hirata K., Murashima M., Umehara N., Tokoroyama T., Hashizume N., Lee W. Y., Takekawa D., Narita K. (2023). Clarification
of the effects of adsorption films of ester-blended oil on friction
by in situ reflectance spectroscopy. Tribiol.
Int..

[11] Dorgham, A. , Neville, A. , Morina, A. , Tribochemistry and Morphology of P-Based Antiwear Films, in Advanced Analytical Methods in Tribology, Dienwiebel, M. ; De Barros Bouchet, M.-I. , Eds.; 2018, Springer International Publishing: Cham. p 159–214.

[12] Cañellas G., Emeric A., Combarros M., Navarro A., Beltran L., Vilaseca M., Vives (2023). Tribological Performance
of Esters, Friction Modifier
and Antiwear Additives for Electric Vehicle Applications. Lubricants.

[13] Zhao R., Mi P., Xu S., Dong S. (2020). Structure
and Properties of Poly-α-olefins
Containing Quaternary Carbon Centers. ACS Omega.

[14] Rudnick, L. R. Synthetics, Mineral Oils, and Bio-Based Lubricants. Chemistry and Technology; Chemical Industries 2020.

[15] Kumar H., Harsha A. P. (2021). Enhanced Lubrication
Ability of Polyalphaolefin and
Polypropylene Glycol by COOH-Functionalized Multiwalled Carbon Nanotubes
as an Additive. Journal of Materials Engineering
and Performance.

[16] Lee C. T., Lee M. B., Hamdan S. H., Chong W. W. F., Chong C. T., Zhang H., Chen A. W. L. (2022). Trimethylolpropane
trioleate as eco-friendly
lubricant additive. Eng. Sci. Technol., Int.
J..

[17] Azman S. S. N., Zulkifli N. W. M., Masjuki H., Gulzar M., Zahid R. (2016). Study of tribological properties
of lubricating oil blend added with
graphene nanoplatelets. J. Mater. Res..

[18] Wang W., Li C., Yang J., Shen Y., Xu J. (2018). Friction performance
of MoDTP and ester-containing lubricants between CKS piston ring and
cast iron cylinder liner. Lubr. Sci..

[19] Jiang S., Li S., Liu L., Wang L., Mominou N. (2015). The tribological properties
and tribochemical analysis of blends of poly alpha-olefins with neopentyl
polyol esters. Tribiol. Int..

[20] Ademi K., Wijanarko W., Espallargas N. (2025). Toward Cost-Effective Environmentally
Acceptable Lubricants: Influence of Synthetic Ester and Low-Viscosity
Polyalphaolefin Blend Type on Additive Performance. Tribol. Lett..

[21] Marmorat T., Wijanarko W., Espallargas N., Khanmohammadi H. (2024). Effect of
the Polar Head Type on the Surface Adsorption and Tribofilm Formation
of Organic Friction Modifiers in Water-Based Lubricants. Langmuir.

[22] Khanmohammadi H., Wijanarko W., Espallargas N. (2023). The role of
tribofilm chemical composition
on wear of austenitic stainless steel lubricated with water-glycol
containing ionic-liquids as additives. Wear.

[23] Matweb , MatWeb: Online Materials Information Resource.

[24] Dawczyk J., Morgan N., Russo J., Spikes H. (2019). Film Thickness and
Friction of ZDDP Tribofilms. Tribol. Lett..

[25] Casasin-Garcia M. L., Mitchell S. G., Espallargas N. (2025). Tungsten-Based
Polyoxometalate-Ionic
Liquid as Lubricant Additive for Low-Viscosity PAO: Effect of Steel
Composition and Microstructure on the Boundary Lubricating Performance. Tribol. Lett..

[26] Wijanarko W., Khanmohammadi H., Espallargas N. (2022). Effect of Steel Hardness and Composition
on the Boundary Lubricating Behavior of Low-Viscosity PAO Formulated
with Dodecanoic Acid and Ionic Liquid Additives. Langmuir.

[27] Pagkalis K., Spikes H., Jelita Rydel J., Ingram M., Kadiric A. (2021). The Influence
of Steel Composition on the Formation and Effectiveness of Anti-wear
Films in Tribological Contacts. Tribol. Lett..

[28] Jelita
Rydel J., Pagkalis K., Kadiric A., Rivera-Díaz-del-Castillo P. E. J. (2017). The
correlation between ZDDP tribofilm morphology and the microstructure
of steel. Tribiol. Int..

[29] Dawczyk J., Russo J., Spikes H. (2019). Ethoxylated
Amine Friction Modifiers
and ZDDP. Tribol. Lett..

