# Supplementary material for: Metal Substrate-Dependent Tribological Performance of Environmentally Acceptable Ester–PAO Lubricant Blends
Source: Langmuir. 2026 Jul 8;42(28):20415–30. doi: 10.1021/acs.langmuir.6c02027 (PMC13394410; doi:10.1021/acs.langmuir.6c02027)
Supplement: Supplementary file 1 [file la6c02027_si_001.pdf]

Supplementary material

Metal Substrate-Dependent Tribological Performance of Environmentally Acceptable Ester–PAO Lubricant Blends

N. Espallargas[1*], K. Ademi[1], W. Wijanarko[1]

The Norwegian Tribology Center, Dept. Mechanical and Industrial Engineering, Norwegian University of Science and Technology – NTNU, Richard Birkelands vei 2b, 7034, Trondheim, Norway.

*corresponding author: nuria.espallargas@ntnu.no

Number of pages: 27

Number of figures: 24

Number of schemes:0

Number of tables: 1

Table of contents

FRICTION AND SPECIFIC WEAR RATE MEASUREMENTS...2

TABLE S1. AVERAGE COEFFICIENT OF FRICTION AND WEAR RATE ALONG WITH STANDARD DEVIATION FOR EACH TEST PERFORMED IN THIS WORK. 2

WEAR TRACK SURFACE CHEMICAL COMPOSITION BY TOF-SIMS 3

FIG. S1 TOF-SIMS NEGATIVE SPECTRA FOR ZDDP IN PAO4 ON STAINLESS STEEL........................4
FIG. S2 TOF-SIMS POSITIVE SPECTRA FOR ZDDP IN PAO4 ON STAINLESS STEEL. 5
FIG. S3 TOF-SIMS NEGATIVE SPECTRA FOR ZDDP IN BLEND ON STAINLESS STEEL. 6
FIG. S4 TOF-SIMS POSITIVE SPECTRA FOR ZDDP IN BLEND ON STAINLESS STEEL.........................7
FIG. S5 TOF-SIMS NEGATIVE SPECTRA FOR ZDDP IN PE ON STAINLESS STEEL. 8
FIG. S6 TOF-SIMS POSITIVE SPECTRA FOR ZDDP IN PE ON STAINLESS STEEL. 9
FIG. S7 TOF-SIMS NEGATIVE SPECTRA FOR ZDDP IN PAO4 ON BEARING STEEL. 10
FIG. S8 TOF-SIMS POSITIVE SPECTRA FOR ZDDP IN PAO4 ON BEARING STEEL.........................11
FIG. S9 TOF-SIMS NEGATIVE SPECTRA FOR ZDDP IN BLEND ON BEARING STEEL. 12
FIG. S10 TOF-SIMS POSITIVE SPECTRA FOR ZDDP IN BLEND ON BEARING STEEL. 13
FIG. S11 TOF-SIMS NEGATIVE SPECTRA FOR ZDDP IN PE ON BEARING STEEL. 14
FIG. S12 TOF-SIMS POSITIVE SPECTRA FOR ZDDP IN PE ON BEARING STEEL............................15
FIG. S13 TOF-SIMS NEGATIVE SPECTRA FOR PEP IN PAO4 ON STAINLESS STEEL.......................16
FIG. S14 TOF-SIMS POSITIVE SPECTRA FOR PEP IN PAO4 ON STAINLESS STEEL.........................17
FIG. S15 TOF-SIMS NEGATIVE SPECTRA FOR PEP IN BLEND ON STAINLESS STEEL. 18
FIG. S16 TOF-SIMS POSITIVE SPECTRA FOR PEP IN BLEND ON STAINLESS STEEL. 19
FIG. S17 TOF-SIMS NEGATIVE SPECTRA FOR PEP IN PE ON STAINLESS STEEL...........................20
FIG. S18 TOF-SIMS POSITIVE SPECTRA FOR PEP IN PE ON STAINLESS STEEL.............................21
FIG. S19 TOF-SIMS NEGATIVE SPECTRA FOR PEP IN PAO4 ON BEARING STEEL. 22
FIG. S20 TOF-SIMS POSITIVE SPECTRA FOR PEP IN PAO4 ON BEARING STEEL. 23
FIG. S21 TOF-SIMS NEGATIVE SPECTRA FOR PEP IN BLEND ON BEARING STEEL........................24
FIG. S22 TOF-SIMS POSITIVE SPECTRA FOR PEP IN BLEND ON BEARING STEEL..........................25
FIG. S23 TOF-SIMS NEGATIVE SPECTRA FOR PEP IN PE ON BEARING STEEL. 26
FIG. S24 TOF-SIMS POSITIVE SPECTRA FOR PEP IN PE ON BEARING STEEL. 27

Supplementary material

Friction and Specific Wear Rate measurements

All tribology tests were performed at least twice. The table below displays the average values for friction and specific wear rate per each test along with the standard deviation (SD).

Table S1. Average coefficient of friction and wear rate along with standard deviation for each test performed in this work.

	Stainless Steel				Bearing Steel			
	**Avg. COF±SD test 1**	**Avg. COF±SD test 2**	**Avg. SWR±SD test 1**	**Avg. SWR ± SD test 2**	**Avg. COF±SD test 1**	**Avg. COF±SD test 2**	**Avg. SWR±SD test 1**	**Avg. SWR±SD test 2**
**PAO4-ZDDP**	0.098± 0.010	0.103± 0.008	2.21E-5± 2.26E-6	3.05E-5± 1.36E-5	0.089± 0.003	0.0089± 0.003	8.1E-6± 2.4E-7	8.5E-6± 5.9E-7
**Blend-ZDDP**	0.103± 0.006	0.104± 0.006	1.7E-5± 7.8E-7	1.4E-5± 1E-6	0.101± 0.002	0.101± 0.001	1E-5± 6.7E-7	1E-5± 1.5E-7
**PE-ZDDP**	0.122± 0.007	0.126± 0.006	3.4E-5± 3.7E-6	4.6E-5± 1E-6	0.109± 0.001	0.116± 0.003	7.4E-6± 1.2E-6	9.3E-6± 6E-7
**PAO4-PEP**	0.134± 0.021	0.128± 0.010	N.D.	N.D.	0.113± 0.004	0.114± 0.005	N.D.	N.D.
**Blend-PEP**	0.108± 0.003	0.107± 0.003	2.96E-5± 1.8E-6	2.53E-5± 1.4E-6	0.077± 0.003	0.078± 0.004	9.52E-6± 4E-7	9.42E-6± 1.1E-7
**PE-PEP**	0.116± 0.005	0.116± 0.004	4.39E-5± 8.7E-7	4.96E-5± 1.5E-6	0.063± 0.003	0.065± 0.002	7.81E-6± 2.4E-7	8.1E-6± 4.6E-7

Supplementary material

Wear track surface chemical composition by ToF-SIMS

Time-of-flight secondary Ion Mass Spectroscopy (ToF-SIMS) was used to analyze the surface chemical composition inside the wear track. The goal was to investigate the adsorption of additives, the tribofilms formed on the surface, and their chemical composition. Positively and negatively charged elements were analyzed, but only the most relevant ones are being plotted in this supplementary material section. The figures below show on the first column the depth profile with the frames-axis representing sequential depth layers of the analyzed surface as it is progressively sputtered during ToF-SIMS analysis. Each frame corresponds to a specific sputtered depth, with intensity values (y-axis) indicating the abundance of secondary ions detected at each layer. The subsequent columns represent the front projection of the depth analysis and the surface map of the depth analysis, respectively. **Figures S1** to **S12** show the ToF-SIMS results of ZDDP in all base lubricants for stainless and bearing steel. **Figures S13** to **S24** show the ToF-SIMS results of PEP in all base lubricants for stainless and bearing steel.

In the case of stainless steel, the negative spectra with the highest intensity correspond to oxygen species (O^- and OH^-), whereas for bearing steel the highest intensity correspond to O^- followed by PO_2^- and S^-. The variations in the intensity depend on the base lubricant and the additive tested.

In the case of the positive spectra, chromium is the most intense specie in stainless steel, whereas iron is the predominant element in bearing steel. In the case of bearing steel, the intensity of iron varies substantially with the type of base lubricant.

Supplementary material

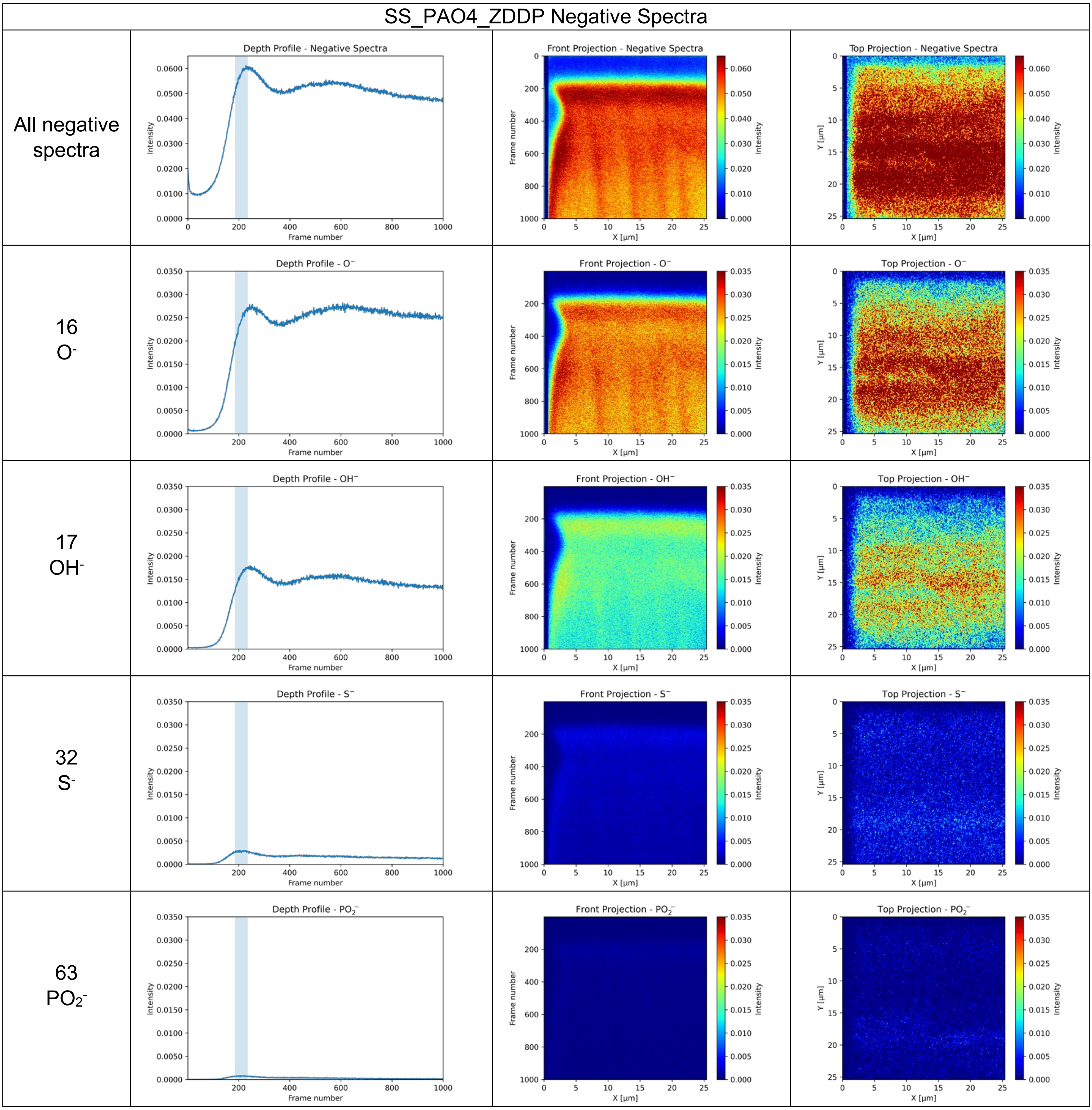

Fig. S1 ToF-SIMS negative spectra for ZDDP in PAO4 on stainless steel.

Supplementary material

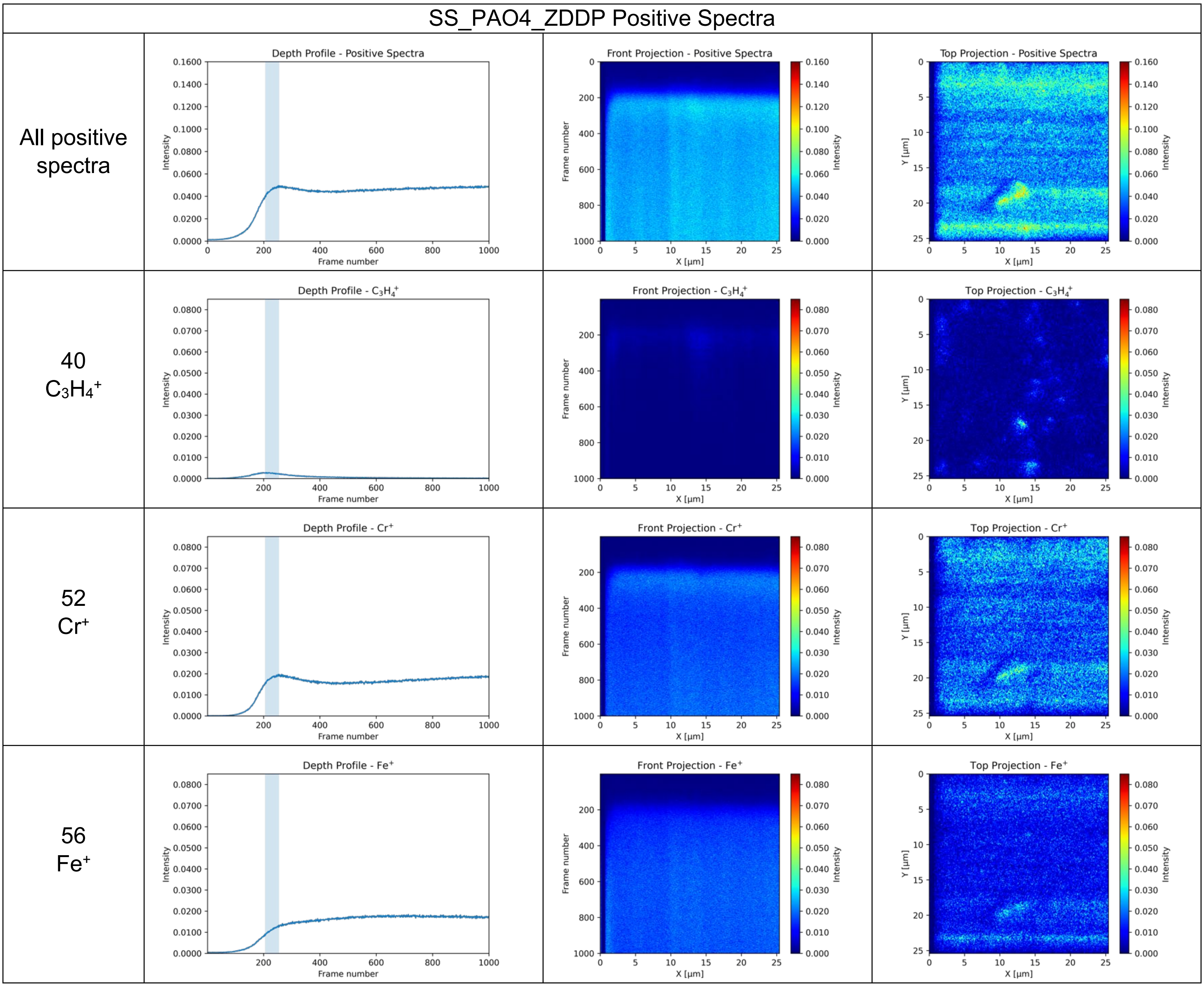

Fig. S2 ToF-SIMS positive spectra for ZDDP in PAO4 on stainless steel.

Supplementary material

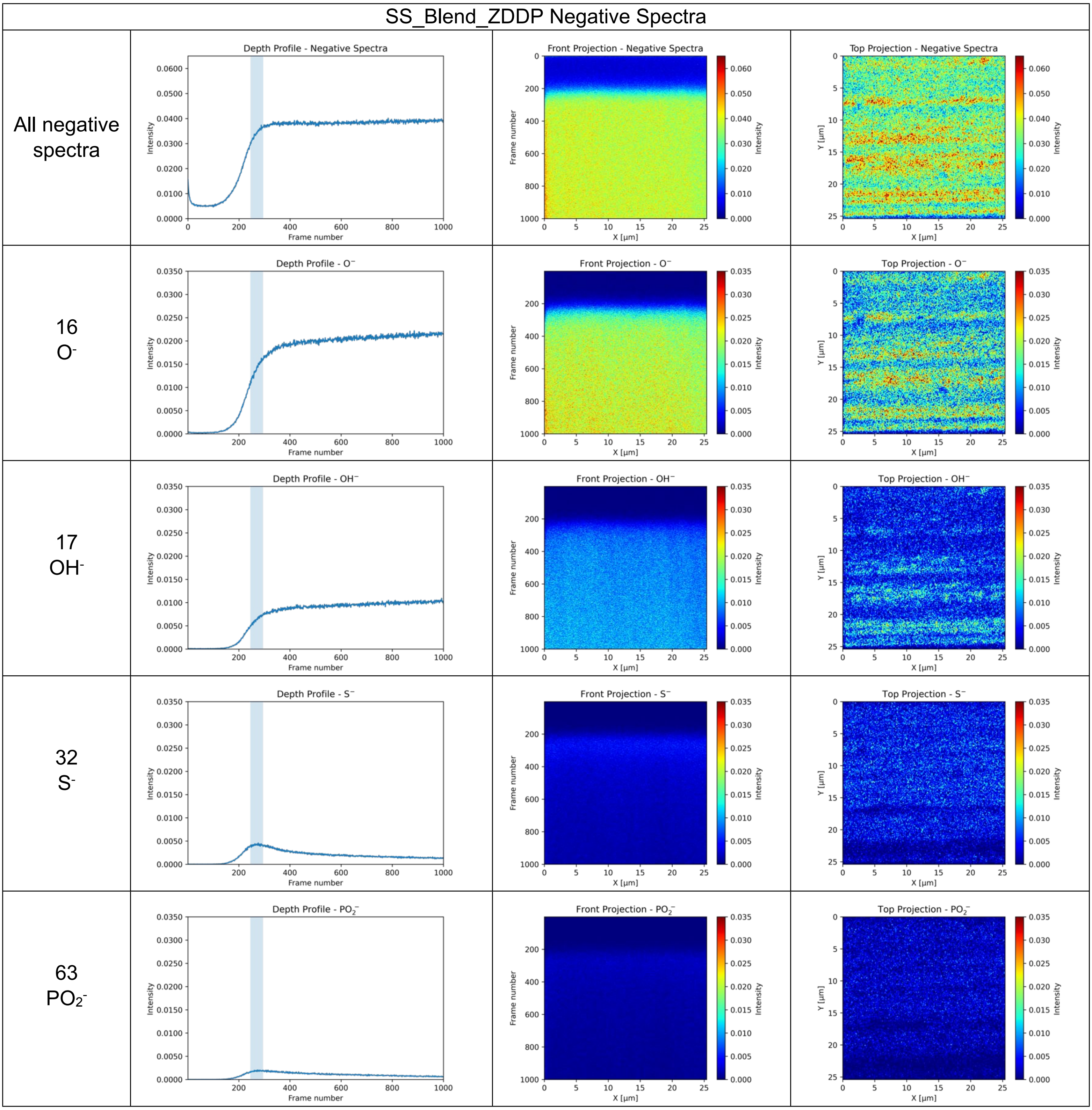

Fig. S3 ToF-SIMS negative spectra for ZDDP in blend on stainless steel.

Supplementary material

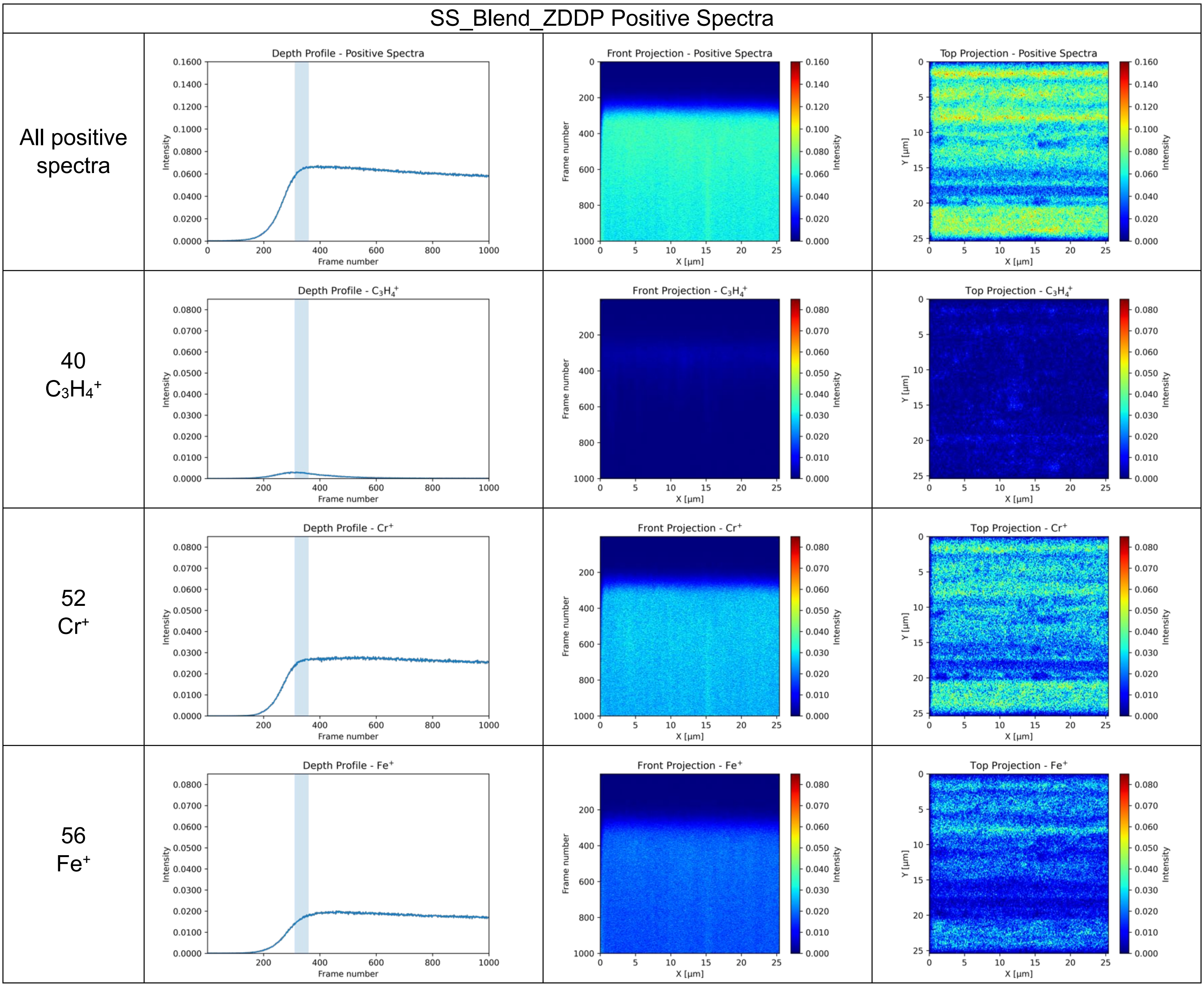

Fig. S4 ToF-SIMS positive spectra for ZDDP in blend on stainless steel.

Supplementary material

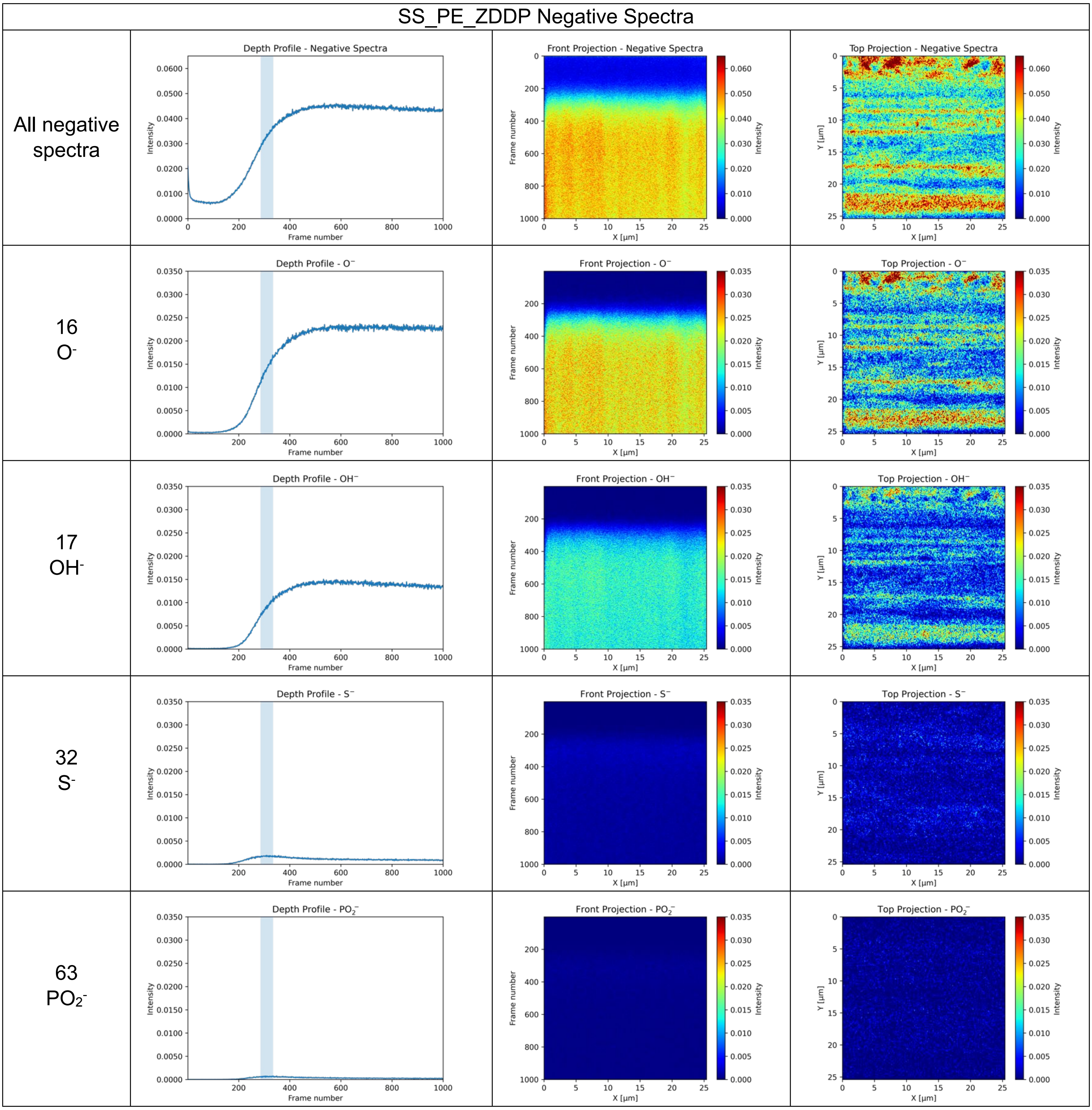

Fig. S5 ToF-SIMS negative spectra for ZDDP in PE on stainless steel.

Supplementary material

<table>
<tr><th colspan="4">SS_PE_ZDDP Positive Spectra</th></tr>
<tr><td>All positive spectra</td><td>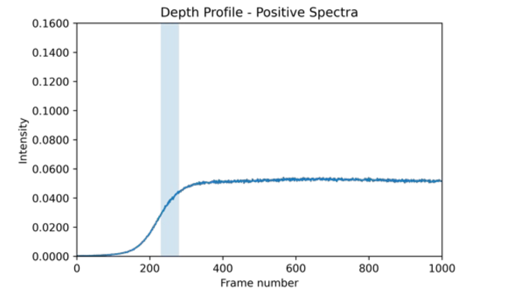</td><td>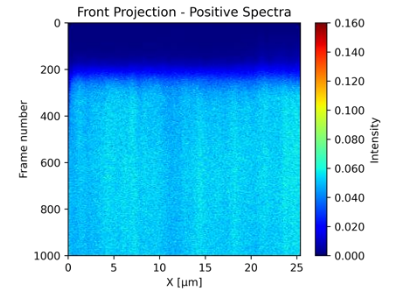</td><td>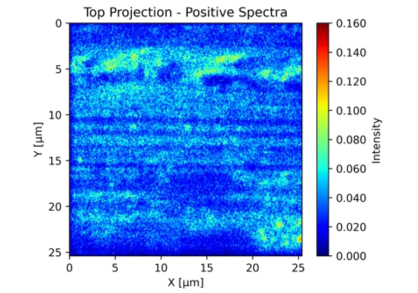</td></tr>
<tr><td>40
$C_3H_4^+$</td><td>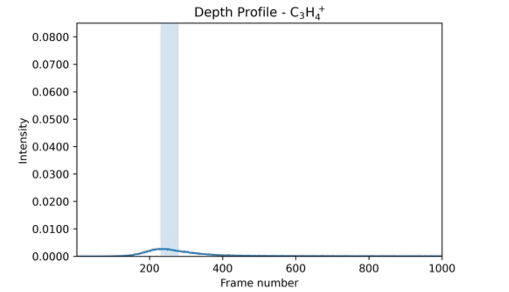</td><td>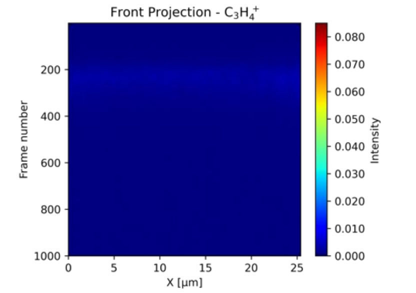</td><td>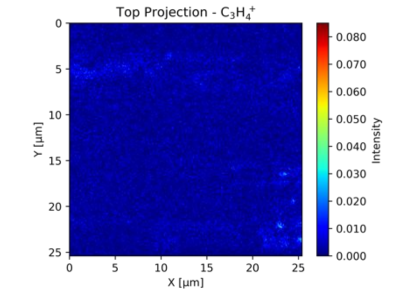</td></tr>
<tr><td>52
Cr^+</td><td>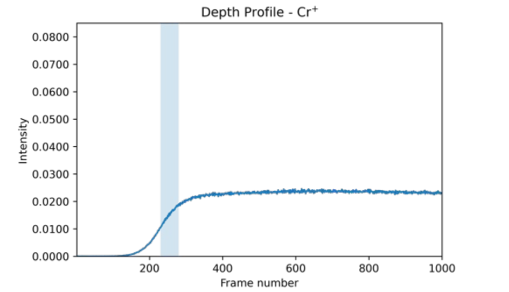</td><td>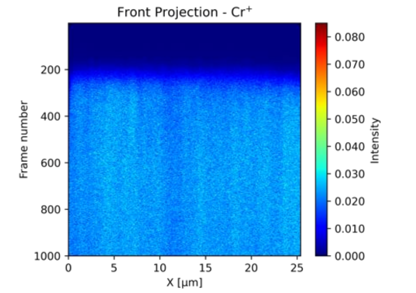</td><td>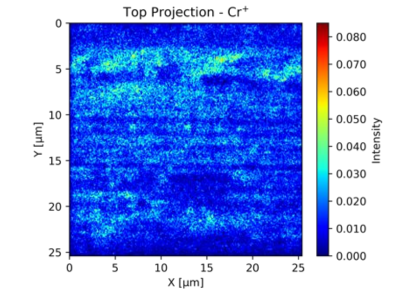</td></tr>
<tr><td>56
Fe^+</td><td>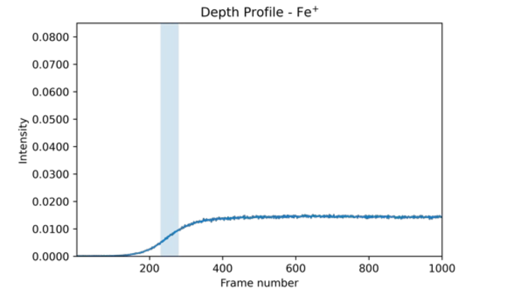</td><td>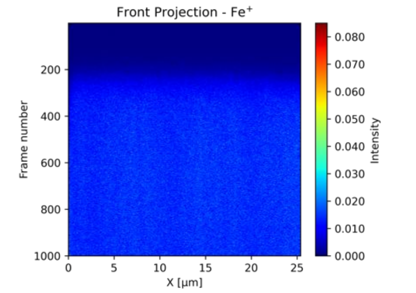</td><td>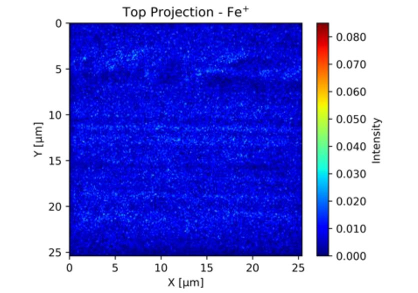</td></tr>
</table>

Fig. S6 ToF-SIMS positive spectra for ZDDP in PE on stainless steel.

Supplementary material

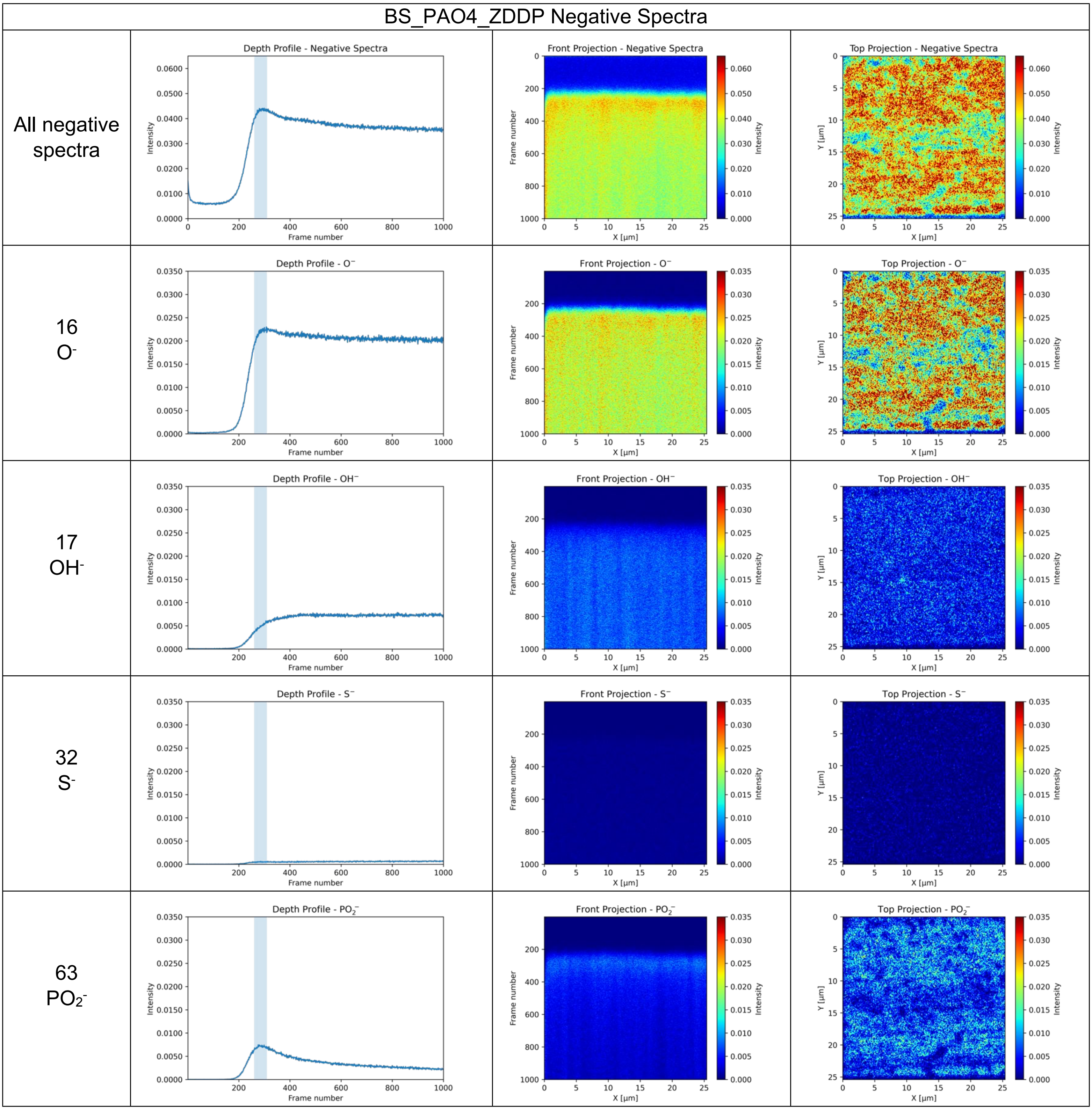

Fig. S7 ToF-SIMS negative spectra for ZDDP in PAO4 on bearing steel.

Supplementary material

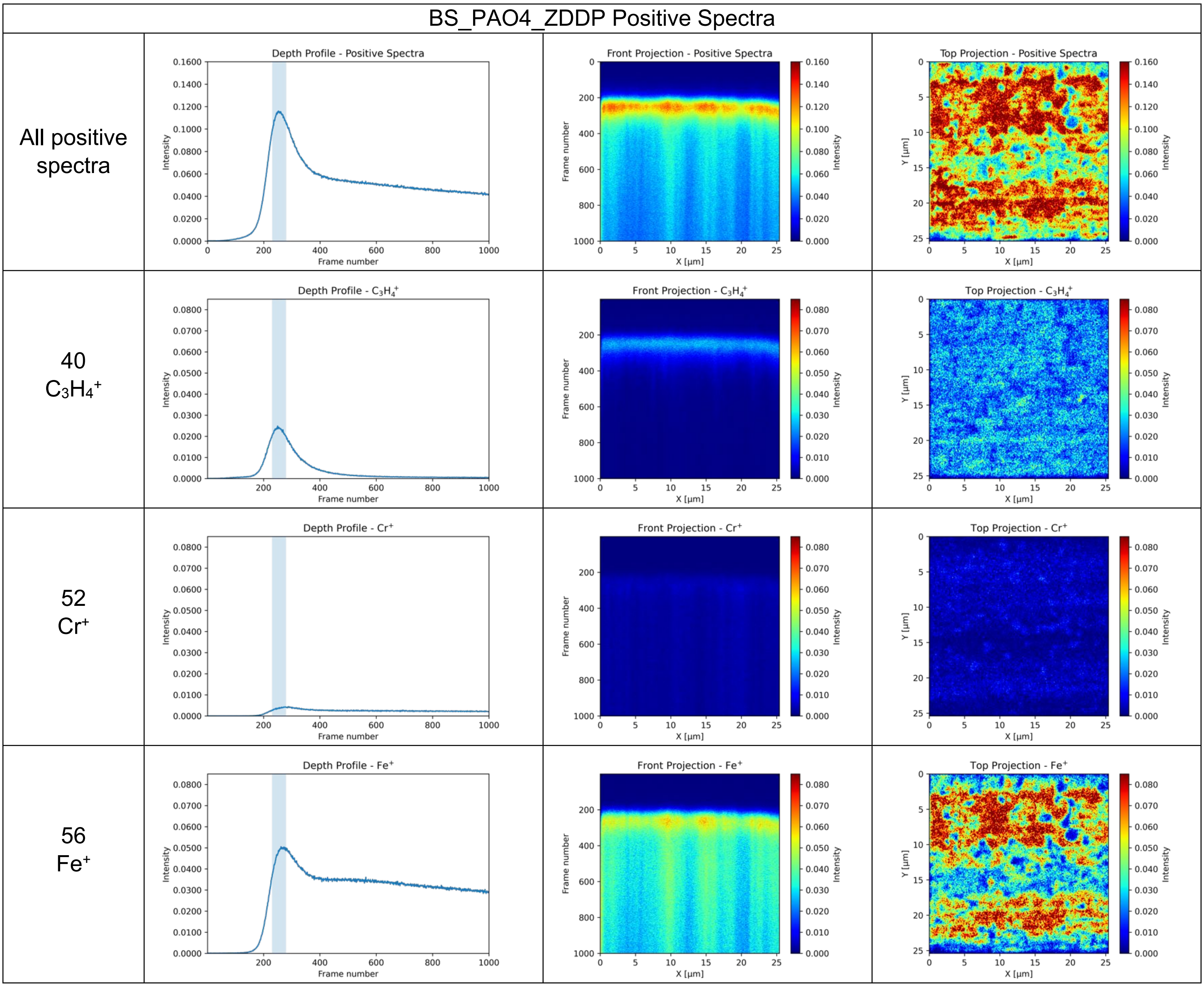

Fig. S8 ToF-SIMS positive spectra for ZDDP in PAO4 on bearing steel.

Supplementary material

BS_Blend_ZDDP Negative Spectra			
All negative spectra	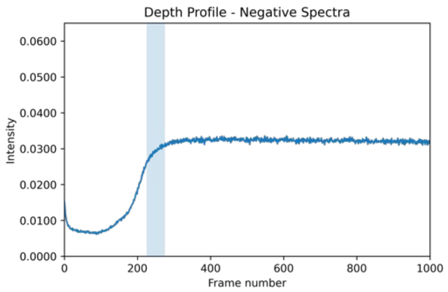	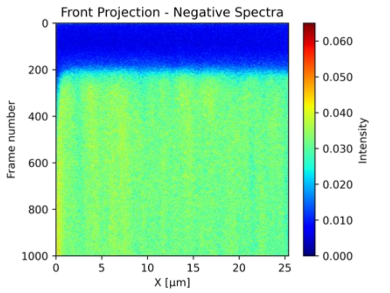	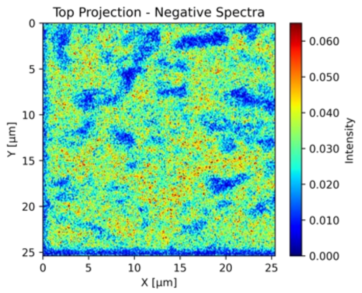
16 O^-	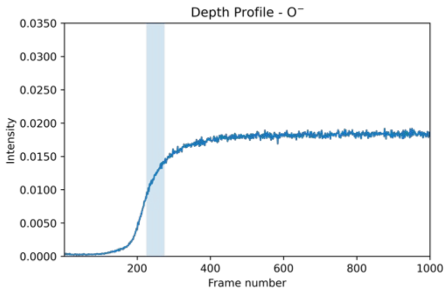	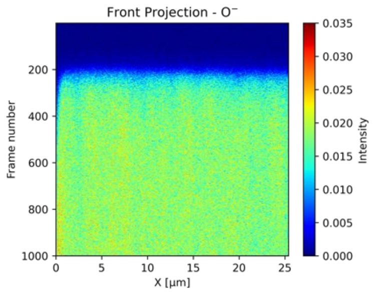	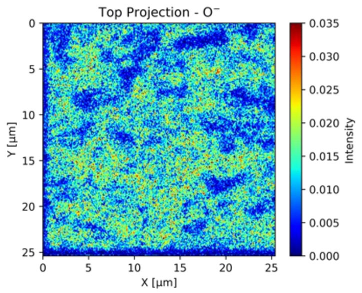
17 OH^-	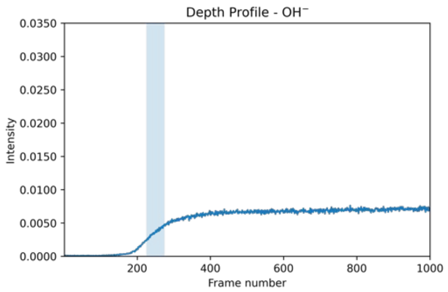	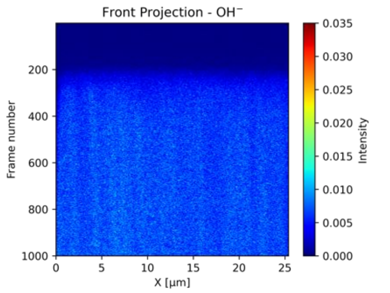	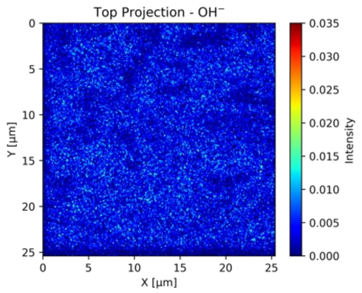
32 S^-	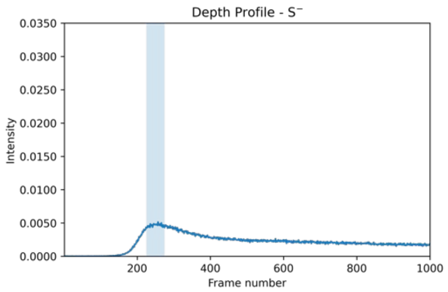	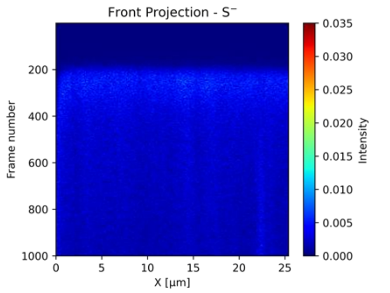	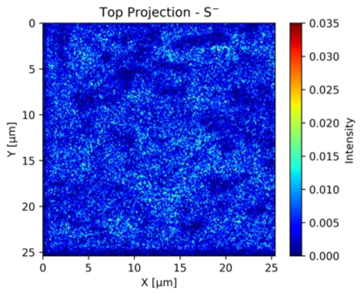
63 PO_2^-	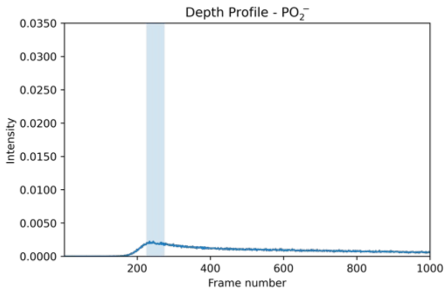	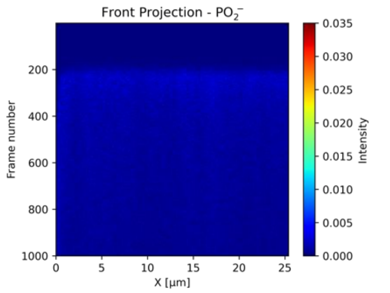	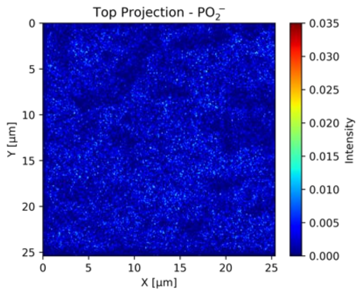

Fig. S9 ToF-SIMS negative spectra for ZDDP in blend on bearing steel.

Supplementary material

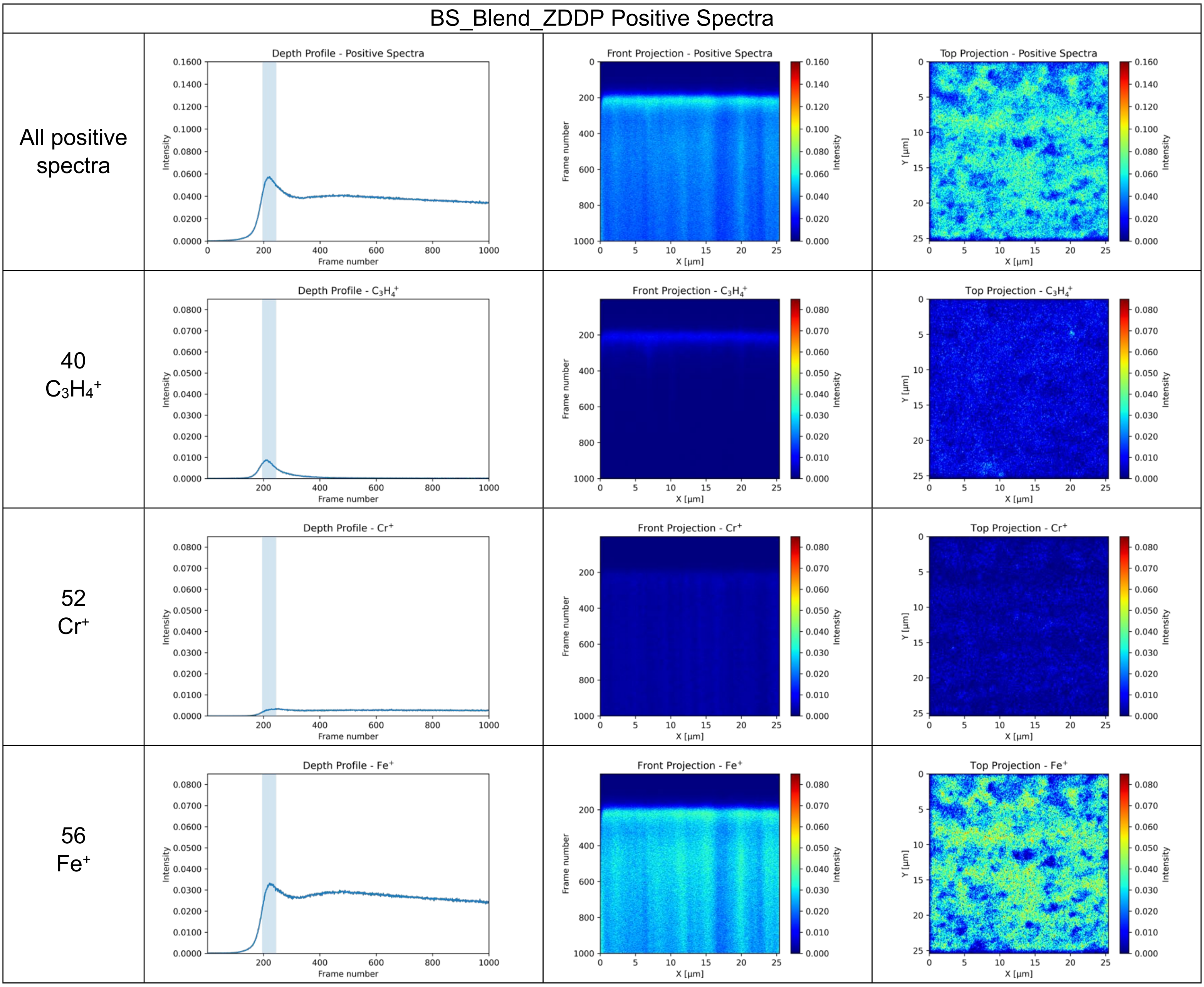

Fig. S10 ToF-SIMS positive spectra for ZDDP in blend on bearing steel.

Supplementary material

BS_PE_ZDDP Negative Spectra			
All negative spectra	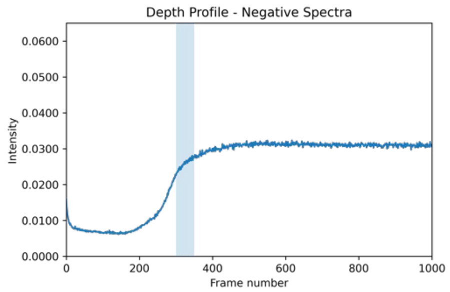	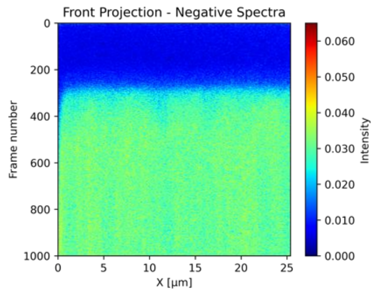	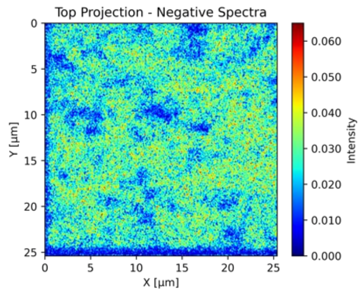
0 16 O^-	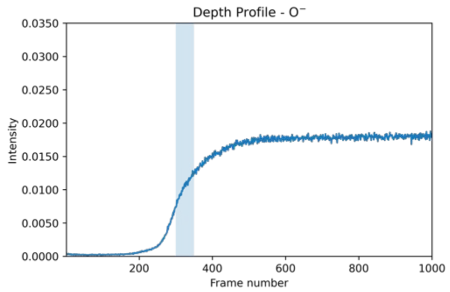	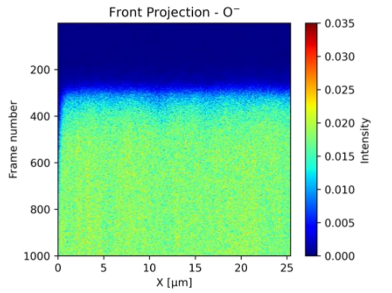	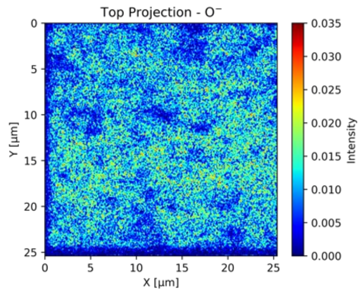
17 OH^-	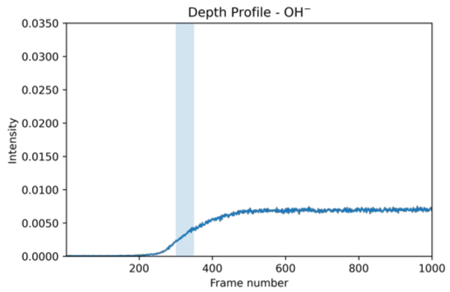	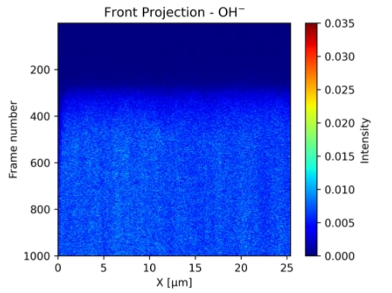	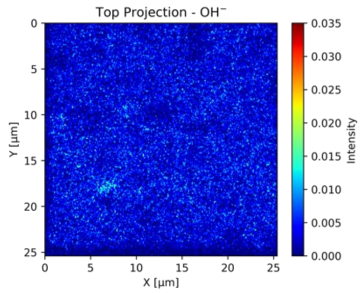
32 S^-	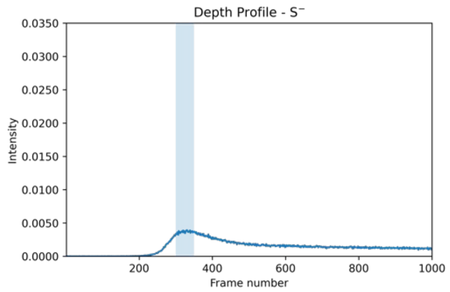	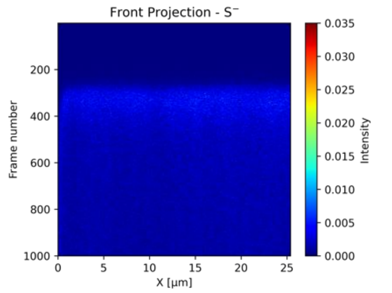	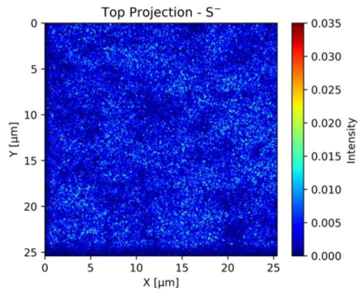
63 PO_2^-	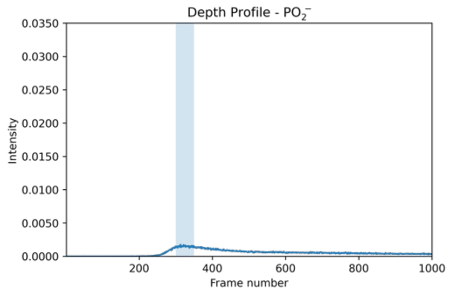	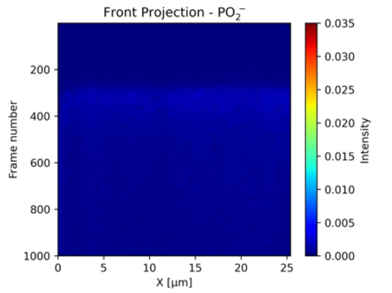	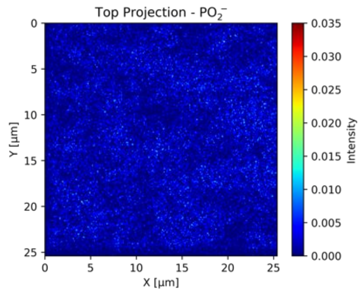

Fig. S11 ToF-SIMS negative spectra for ZDDP in PE on bearing steel.

Supplementary material

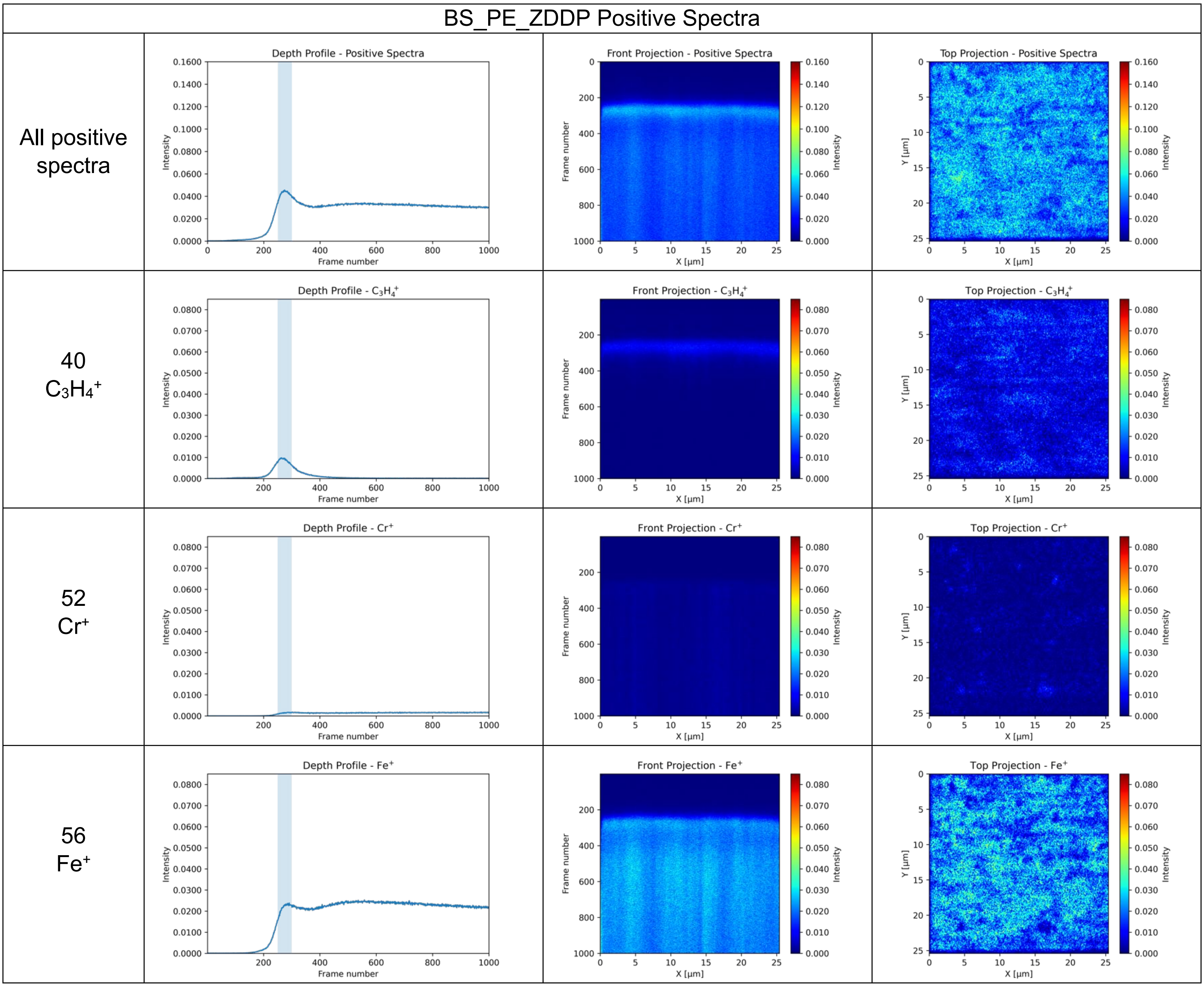

Fig. S12 ToF-SIMS positive spectra for ZDDP in PE on bearing steel.

Supplementary material

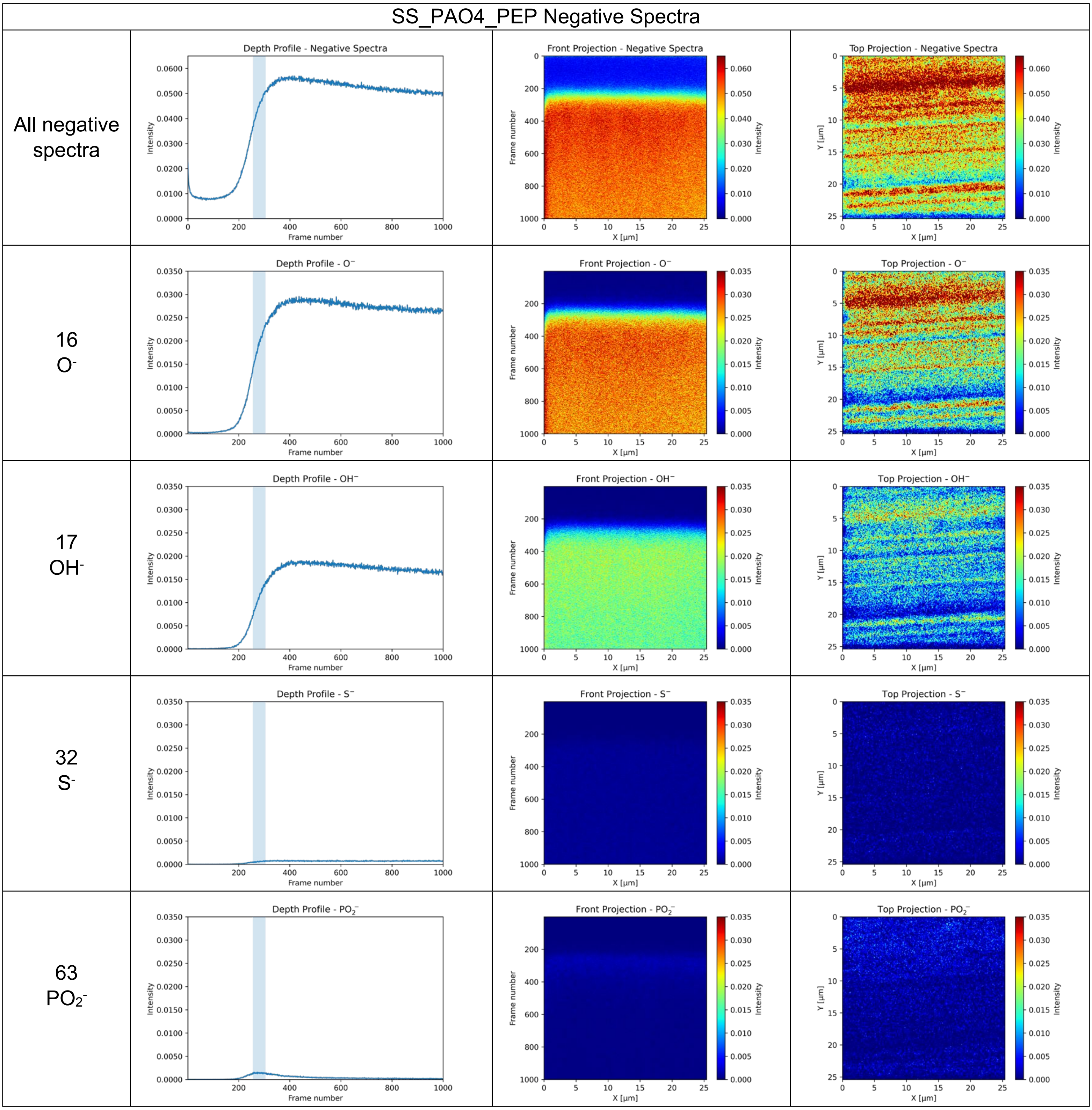

Fig. S13 ToF-SIMS negative spectra for PEP in PAO4 on stainless steel.

Supplementary material

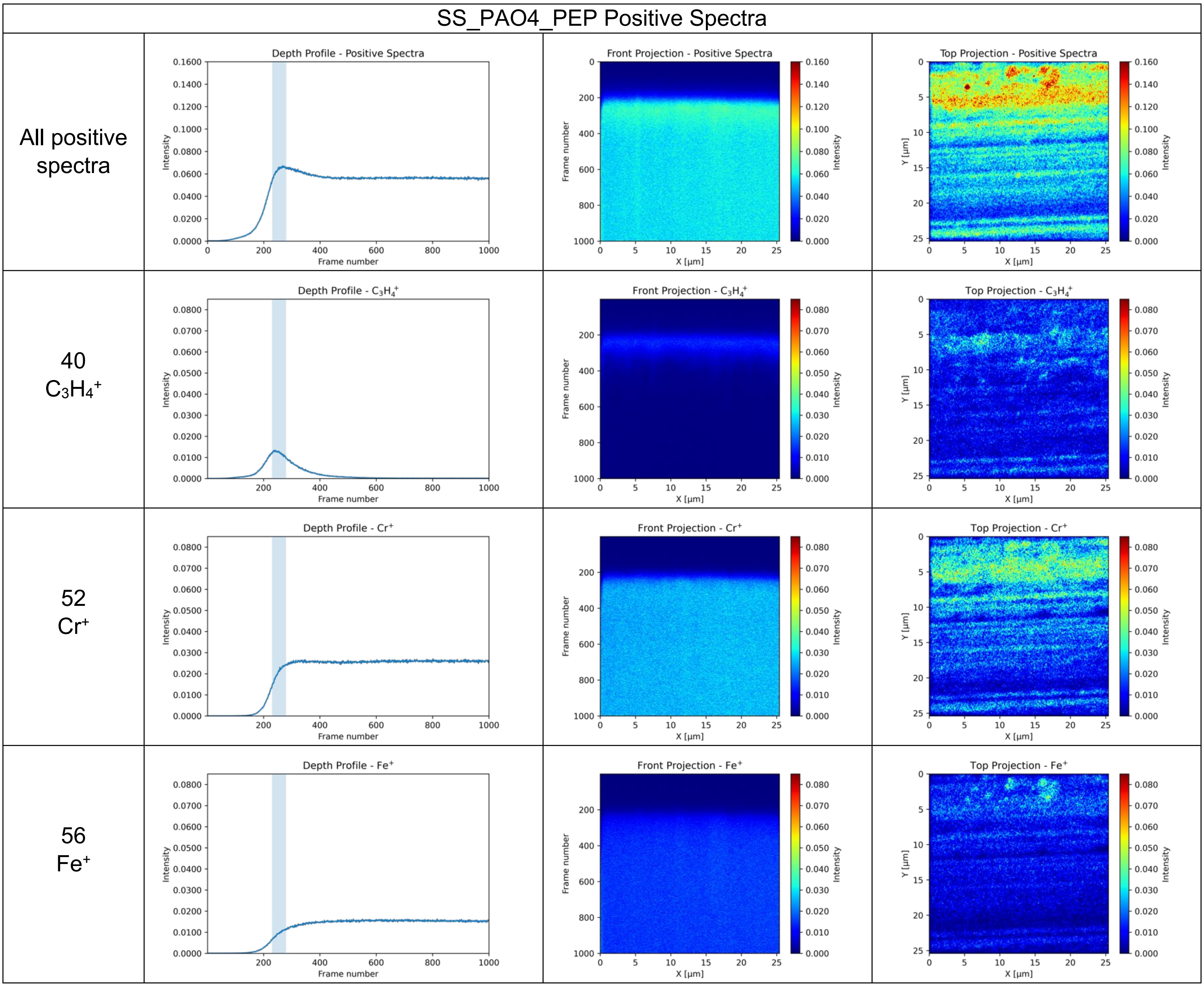

Fig. S14 ToF-SIMS positive spectra for PEP in PAO4 on stainless steel.

Supplementary material

SS_Blend_PEP Negative Spectra			
All negative spectra	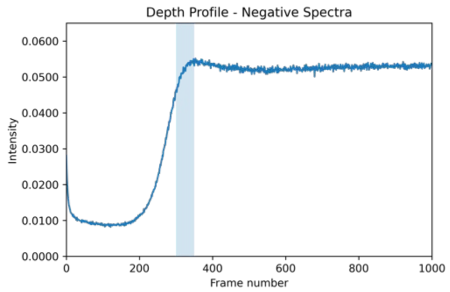	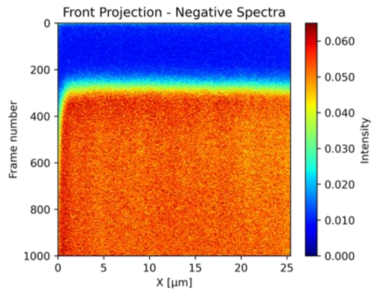	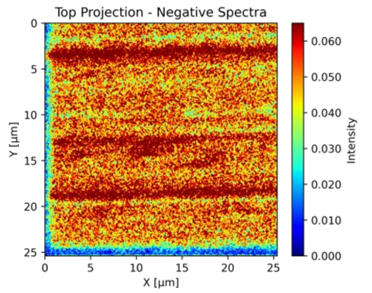
16 O^-	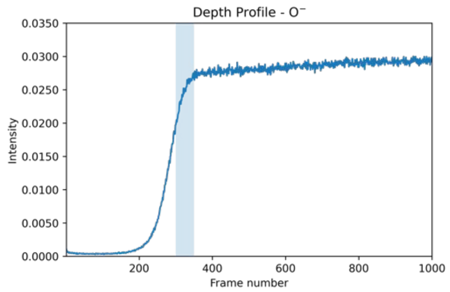	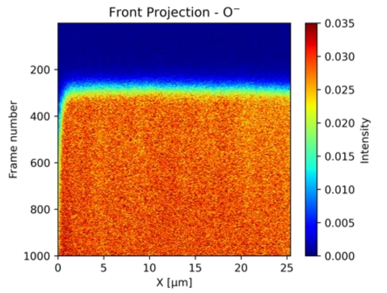	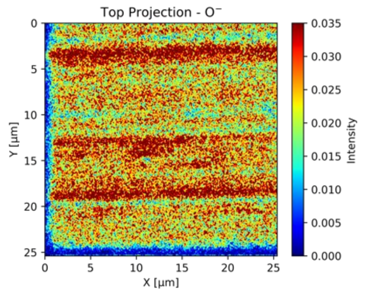
17 OH^-	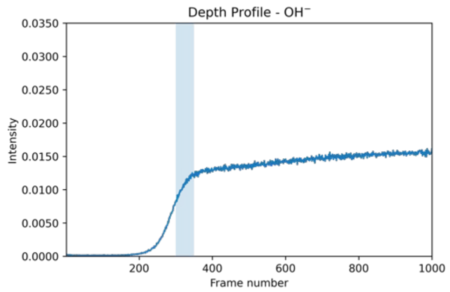	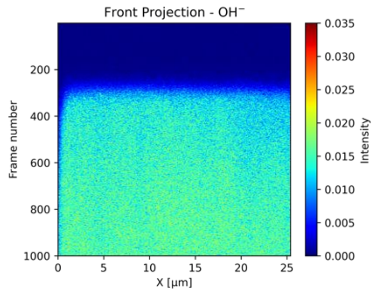	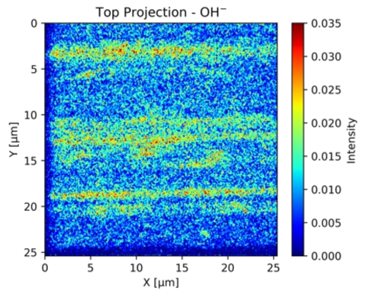
32 S^-	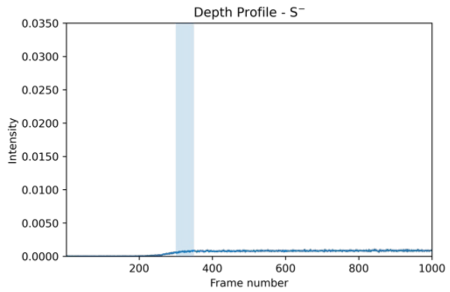	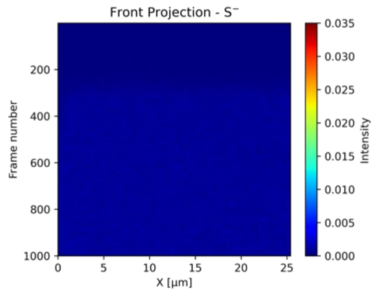	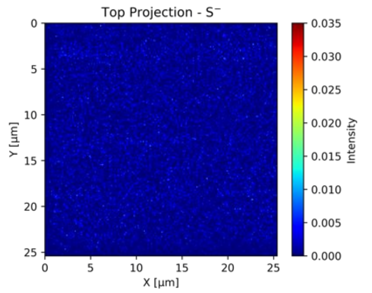
63 PO_2^-	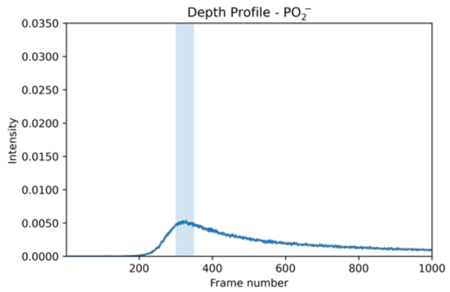	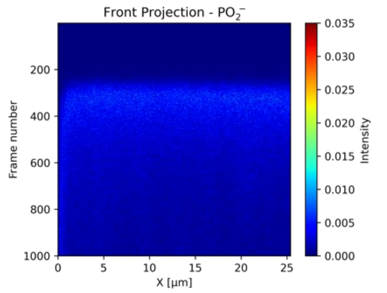	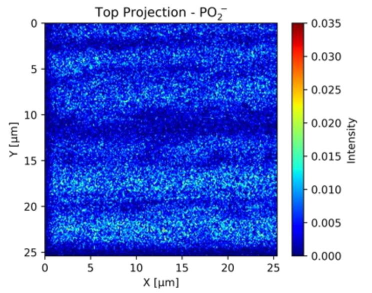

Fig. S15 ToF-SIMS negative spectra for PEP in blend on stainless steel.

Supplementary material

SS_Blend_PEP Positive Spectra			
All postive spectra	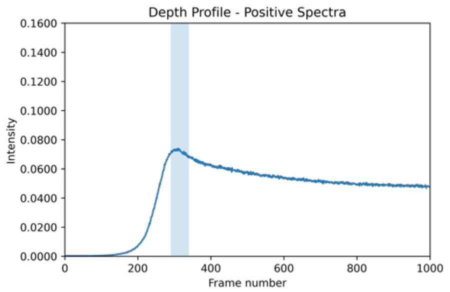	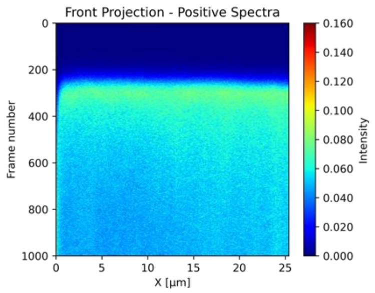	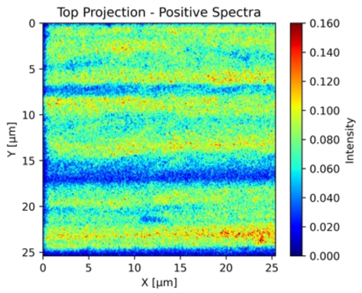
40 $C_3H_4^+$	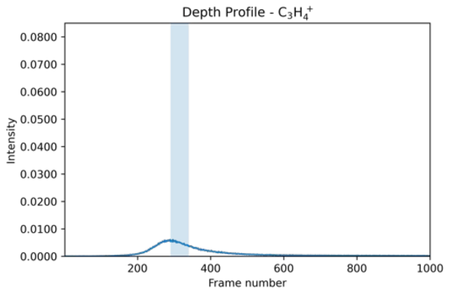	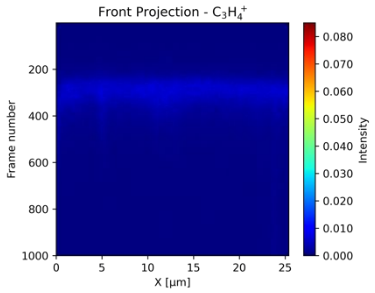	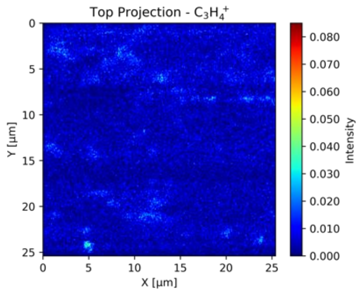
52 Cr^+	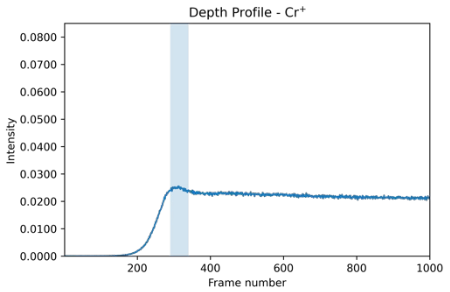	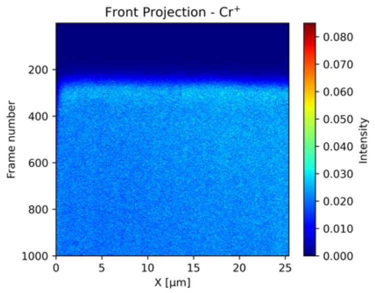	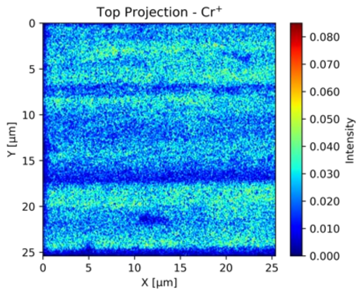
56 Fe^+	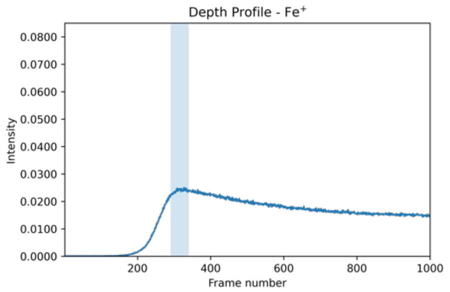	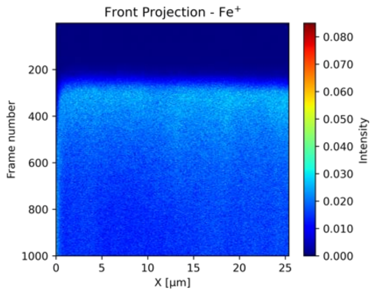	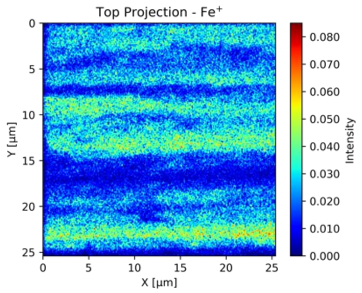

Fig. S16 ToF-SIMS positive spectra for PEP in blend on stainless steel.

Supplementary material

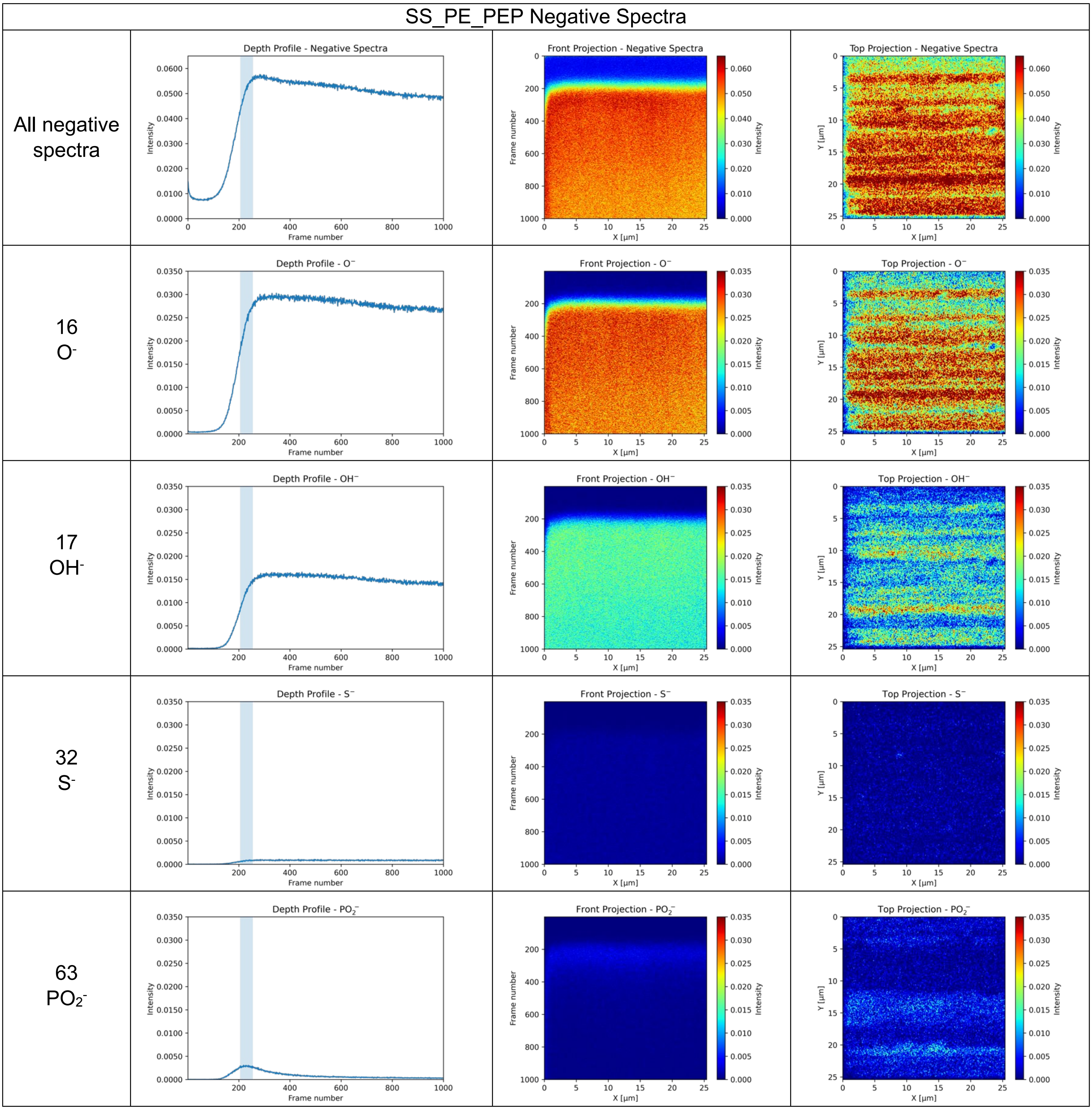

Fig. S17 ToF-SIMS negative spectra for PEP in PE on stainless steel.

Supplementary material

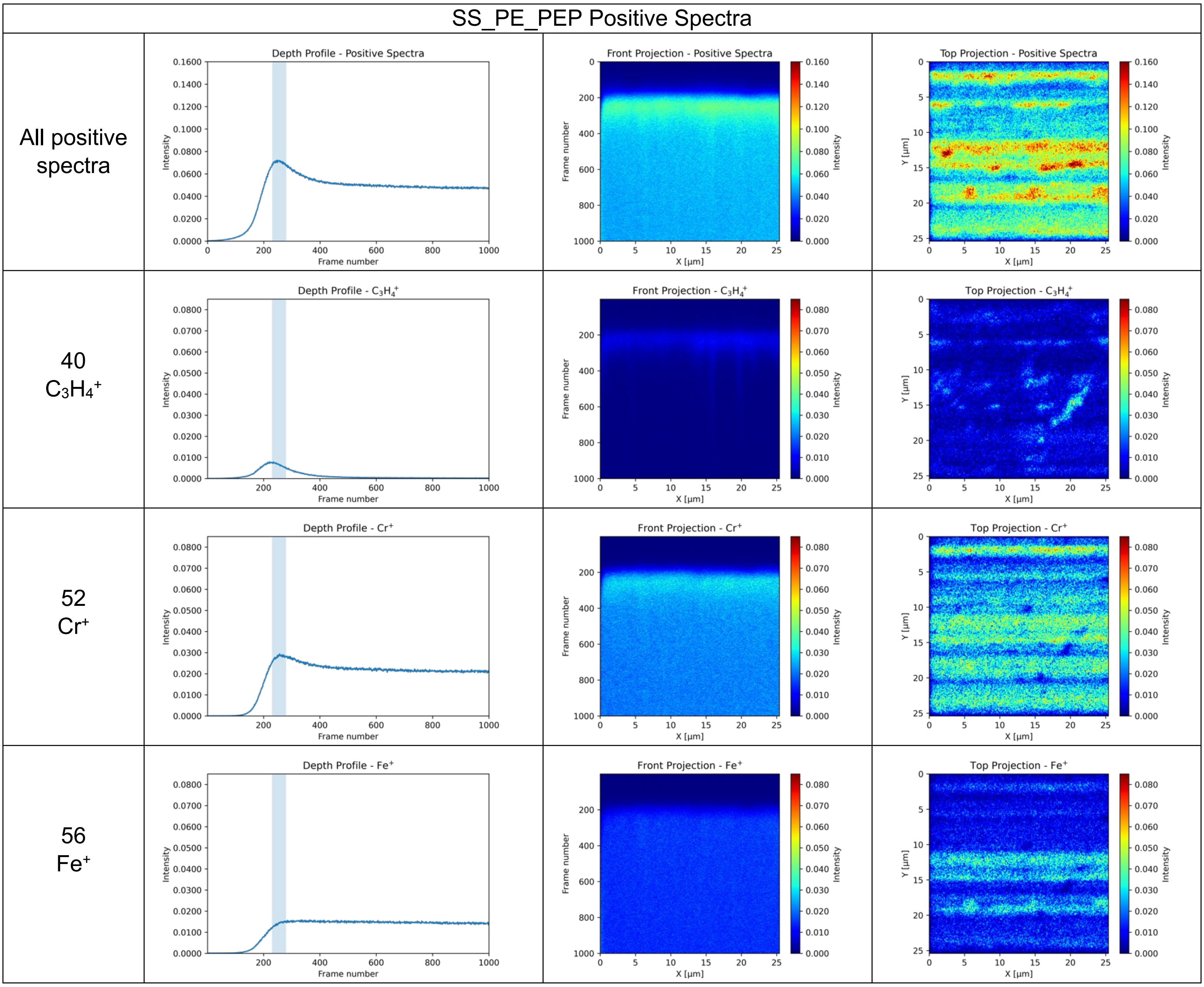

Fig. S18 ToF-SIMS positive spectra for PEP in PE on stainless steel.

Supplementary material

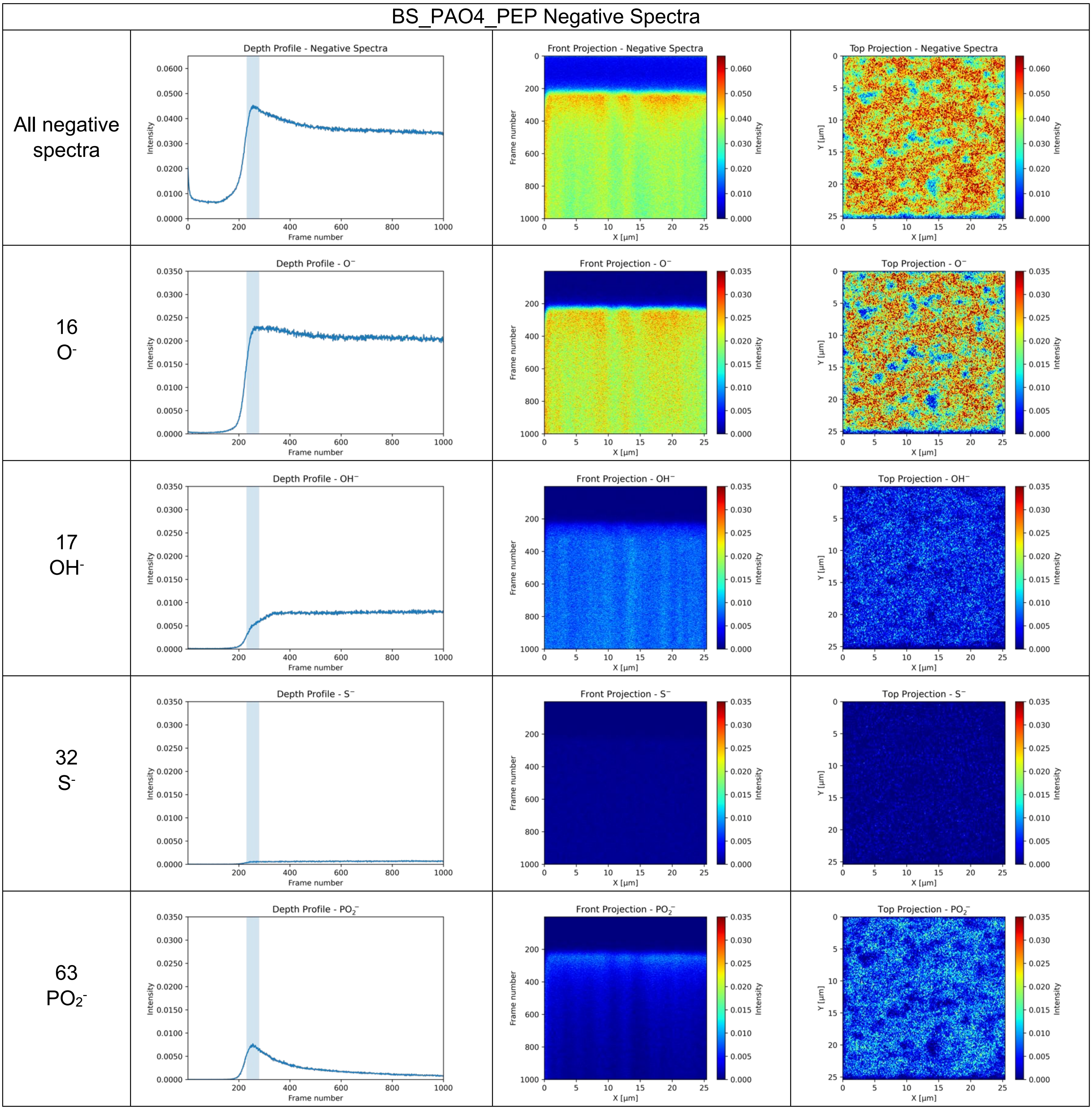

Fig. S19 ToF-SIMS negative spectra for PEP in PAO4 on bearing steel.

Supplementary material

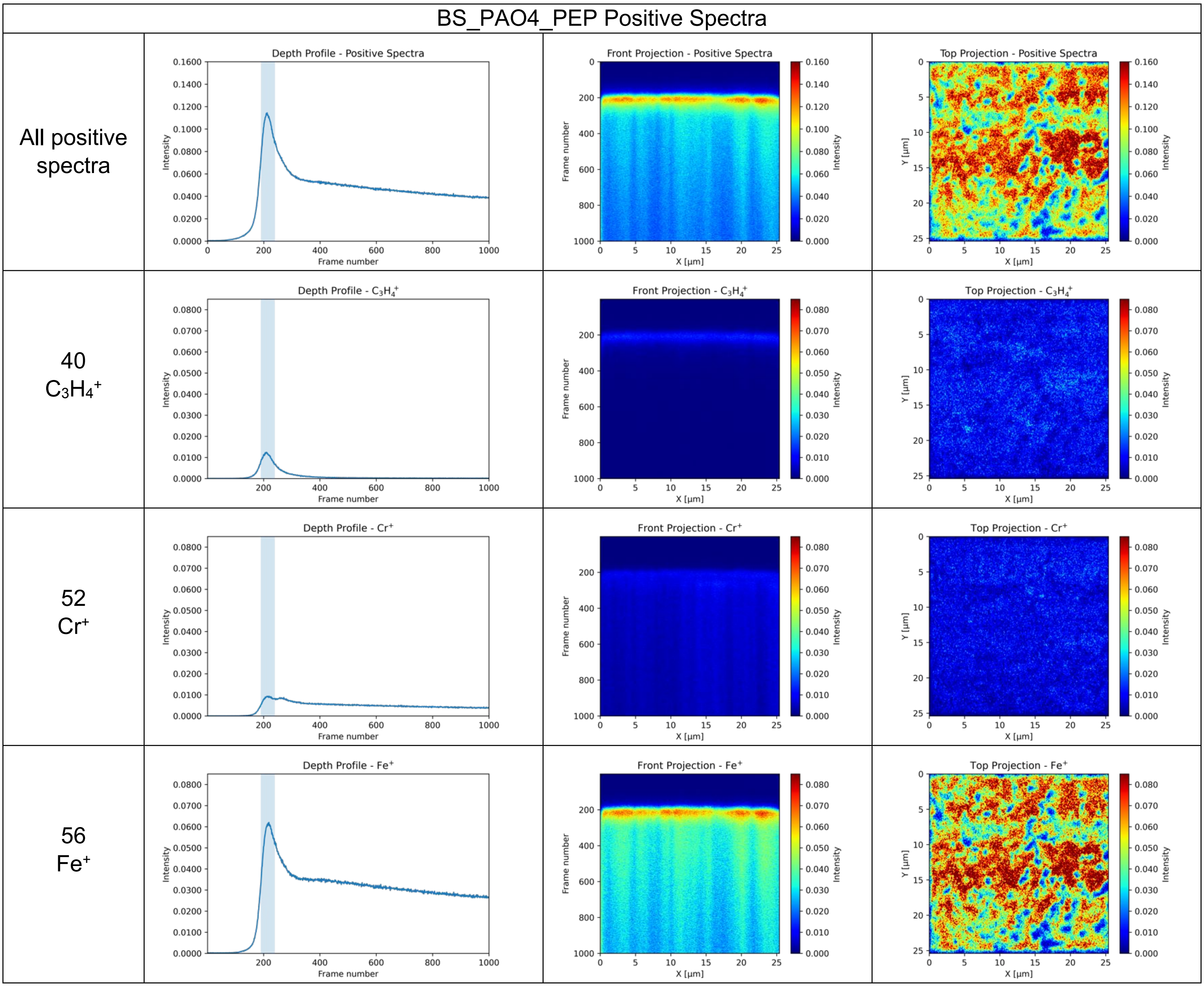

Fig. S20 ToF-SIMS positive spectra for PEP in PAO4 on bearing steel.

Supplementary material

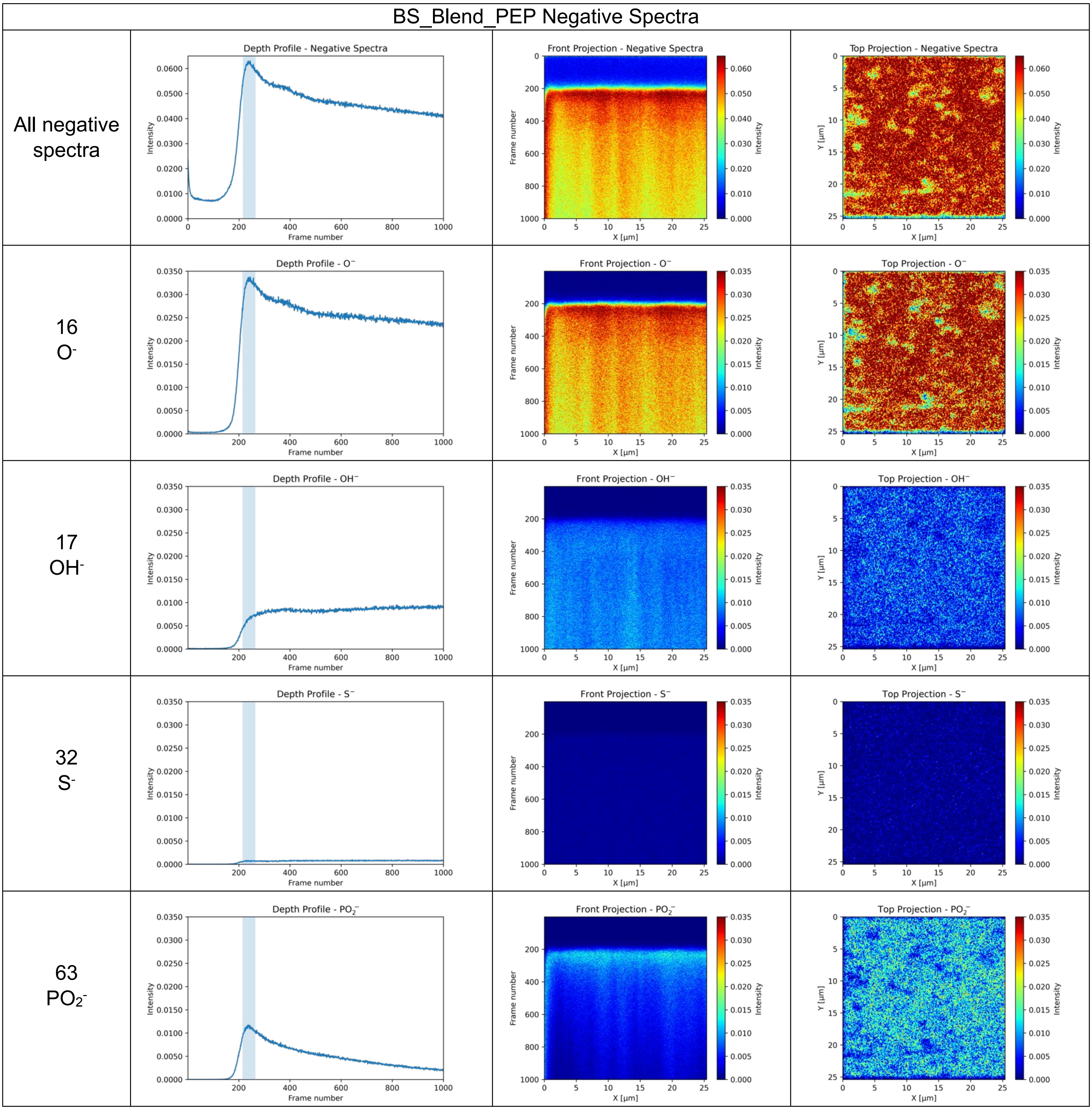

Fig. S21 ToF-SIMS negative spectra for PEP in blend on bearing steel.

Supplementary material

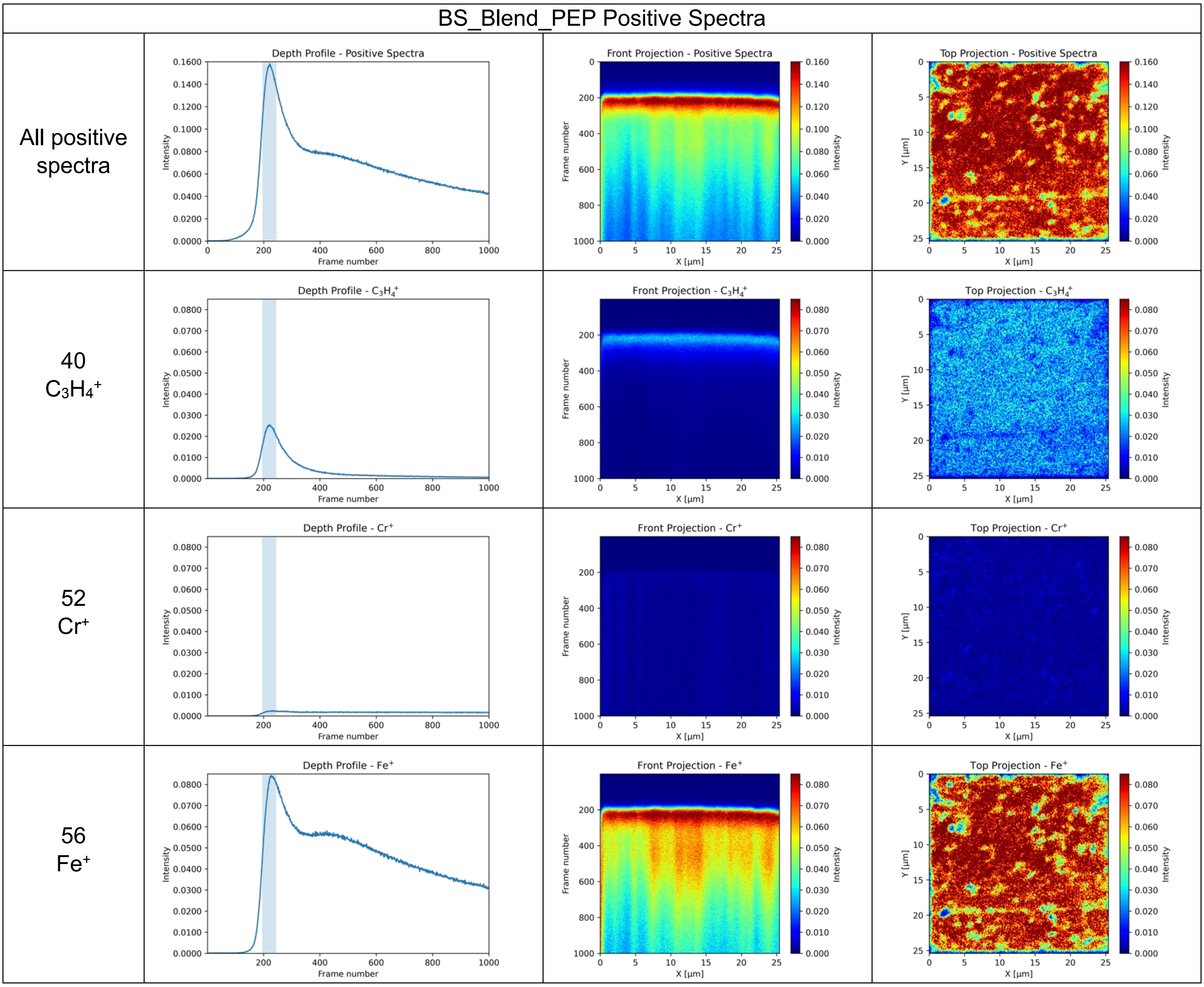

Fig. S22 ToF-SIMS positive spectra for PEP in blend on bearing steel.

Supplementary material

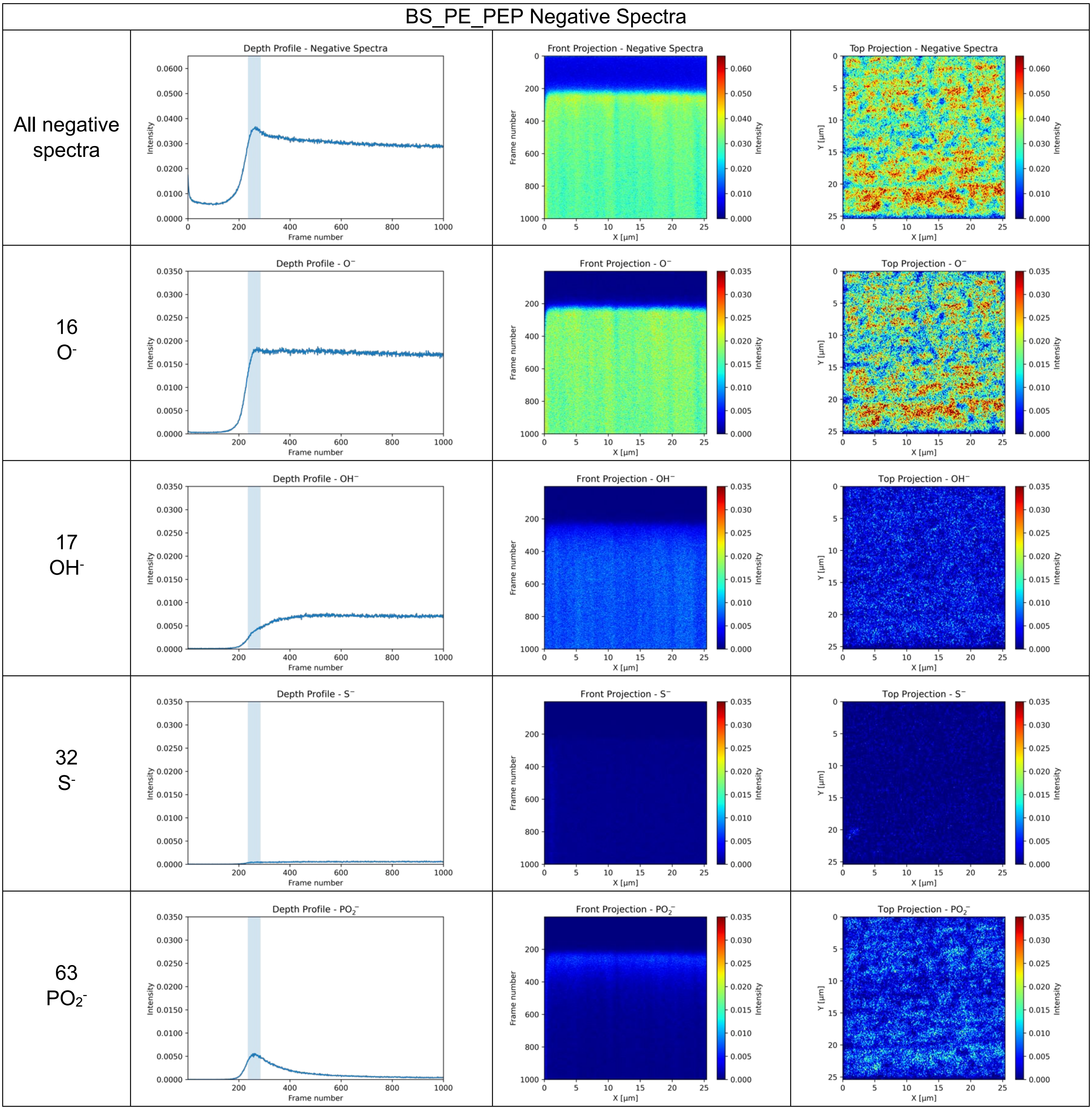

Fig. S23 ToF-SIMS negative spectra for PEP in PE on bearing steel.

Supplementary material

BS_PE_PEP Positive Spectra			
All positive spectra	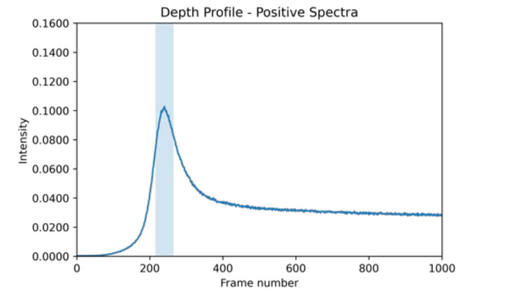	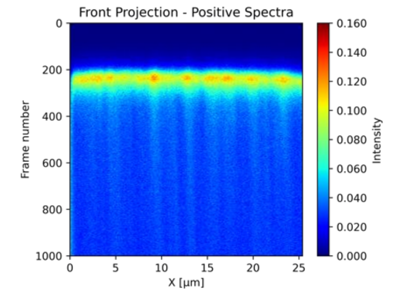	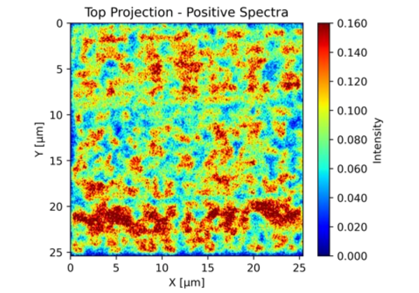
40 $C_3H_4^+$	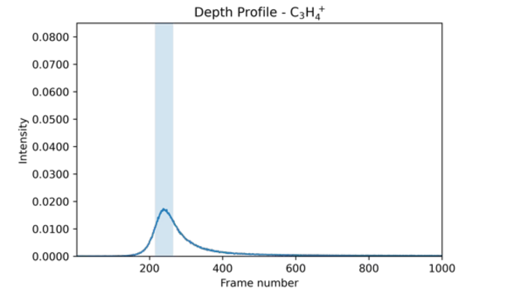	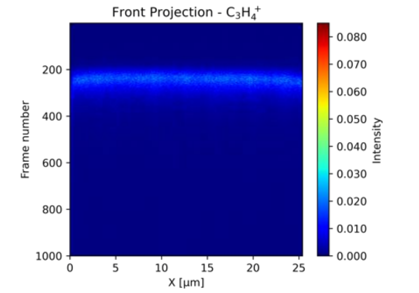	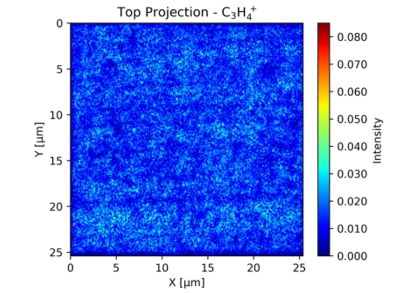
52 Cr^+	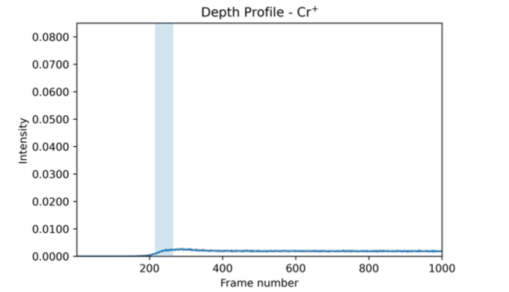	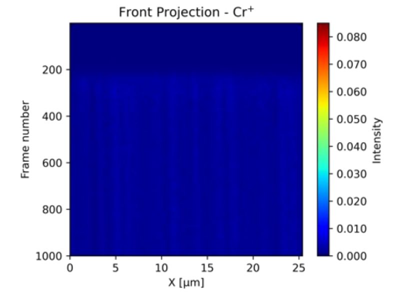	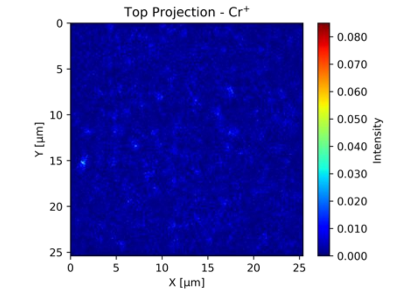
56 Fe^+	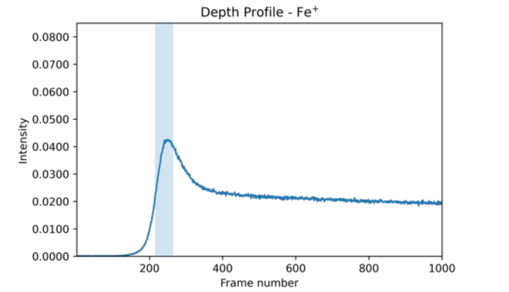	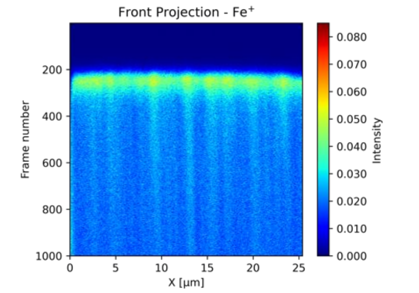	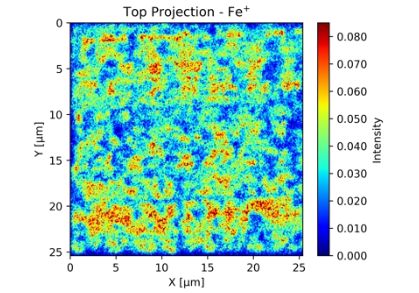

Fig. S24 ToF-SIMS positive spectra for PEP in PE on bearing steel.
